# Evaluation of Trials Comparing Single-Enantiomer Drugs to Their Racemic Precursors: A Systematic Review

**DOI:** 10.1001/jamanetworkopen.2021.5731

**Published:** 2021-05-06

**Authors:** Aaron S. Long, Audrey D. Zhang, Caitlin E. Meyer, Alexander C. Egilman, Joseph S. Ross, Joshua D. Wallach

**Affiliations:** 1 Yale University School of Medicine, New Haven, Connecticut; 2 Duke University Health System, Durham, North Carolina; 3 Harvey Cushing/John Hay Whitney Medical Library, Yale University, New Haven, Connecticut; 4 Center for Outcomes Research and Evaluation, New Haven, Connecticut; 5 Section of General Internal Medicine, Yale School of Medicine, New Haven, Connecticut; 6 National Clinician Scholars Program, Yale School of Medicine, Department of Internal Medicine, New Haven, Connecticut; 7 Department of Health Policy and Management, Yale School of Public Health, New Haven, Connecticut; 8 Department of Environmental Health Sciences, Yale School of Public Health, New Haven, Connecticut

## Abstract

**Importance:**

Chiral switching, a strategy in which drug manufacturers develop a single-enantiomer formulation of a drug to be substituted for a racemic formulation, allows manufacturers to maintain market exclusivity for drugs losing patent protection, even without demonstrating superior efficacy or safety.

**Objective:**

To identify and characterize all randomized clinical trials (RCTs) directly comparing a Food and Drug Administration (FDA)–approved single-enantiomer drug against a previously approved racemic drug for 1 or more efficacy or safety end points.

**Evidence Review:**

Drugs were identified using the Drugs@FDA database. Randomized clinical trials were identified using Ovid MEDLINE (1949 to October 22, 2019), Ovid Embase (1974 to October 22, 2019), Web of Science Core Collection (all years), ClinicalTrials.gov, and Cochrane Central Registry of Controlled Trials (CENTRAL, Wiley, Issue 8 of 12, October 22, 2019). Trials were characterized as favoring the single-enantiomer or racemic drugs based on whether the primary efficacy, secondary efficacy, and safety end points achieved each study’s defined significance level (eg, *P* < .05). Trials were characterized as favoring neither drug if no statistically significant differences were reported for any end point or if both drugs were found to be superior for 1 or more separate end points.

**Findings:**

Fifteen FDA-approved single-enantiomer drugs were identified with racemic precursors approved in the US or Europe. For 3 single-enantiomer racemic drug pairs, no RCTs directly comparing the drugs were identified. For the remaining 12 pairs, 185 RCTs comparing efficacy or safety of the drug pairs were identified, 124 (67.0%) of which studied 1 pair (levobupivacaine/bupivacaine). There were 179 RCTs directly comparing drug pairs using efficacy end points, of which 23 (12.8%) favored the single enantiomer based on primary efficacy end point results. There were 124 RCTs directly comparing drug pairs using safety end points, of which 17 (13.7%) favored the single-enantiomer drug. For 9 of the 15 single-enantiomer drugs (60.0%), no RCTs were identified providing evidence of improved efficacy, based on primary end point results, or safety as compared with their racemic precursors.

**Conclusions and Relevance:**

The results of this systematic review suggest that most newly marketed FDA-approved single-enantiomer drugs are infrequently directly compared with their racemic precursors, and when compared, they are uncommonly found to provide improved efficacy or safety, despite their greater costs.

## Key Points

**Question:**

How often do randomized clinical trials directly compare new single-enantiomer drugs to their existing racemic precursors, and how often are efficacy or safety differences observed?

**Findings:**

In this systematic review of 15 single-enantiomer racemic drug pairs, 185 direct-comparison randomized clinical trials (median, 2 trials; interquartile range, 1-8 trials) were identified, 124 (67.0%) of which studied 1 drug pair. For 9 single-enantiomer drugs, no randomized clinical trials were identified providing evidence of improved efficacy, based on primary end point results, or safety as compared with their racemic precursors.

**Meaning:**

Results of this systematic review suggest that most newly marketed single-enantiomer drugs are infrequently directly compared with their existing racemic precursors, and when compared, they are uncommonly found to provide improved efficacy or safety, despite their greater costs.

## Teaser

This systematic review uses data from randomized clinical trials to investigate how often new single-enantiomer drugs are compared with their existing racemic precursors and determine whether safety or efficacy differences are observed.

## Introduction

In the US, a system of patents and market exclusivity provides manufacturers of new drugs protection against competition from generic drugs.^1^ Although this system balances higher prices from delayed generic competition with the need to promote new drug innovation,^2^ certain strategies, such as “chiral switching,” can allow manufacturers to maintain market exclusivity on drugs losing patent protection.^3,4^ Chiral drugs are made of mirror-image molecules called enantiomers (eg, a 50:50 racemic mixture of enantiomers), and in chiral switching, manufacturers develop a single-enantiomer drug that can be substituted for the already-approved racemic version (eg, escitalopram [(*S*)-citalopram] for citalopram [(*R*)- and (*S*)-citalopram]).^3,5,6^ Although manufacturers must submit a New Drug Application to the Food and Drug Administration (FDA) for these single-enantiomer formulations,^7,8,9^ concerns have been raised about whether these new drugs show sufficient evidence of superior efficacy or safety to justify their costs.^10^

The theoretical benefit of chiral switching is based in different biological activities of enantiomers.^3,5,11^ For instance, the single-enantiomer formulation of racemic albuterol, (*R*)-albuterol, is believed to be responsible for its therapeutic effect and has a superior pharmacodynamic profile to (*S*)-albuterol.^3^ However, the suggested benefits of single-enantiomer drugs are often based on nonclinical trial evidence, such as in vitro and animal studies.^3,5,12,13,14^ Moreover, manufacturers of single-enantiomer drugs are not required to conduct randomized clinical trials (RCTs) directly comparing their products with existing racemic drugs before receiving FDA approval.^15,16,17^ Between 2001 and 2011, only one-third of approvals of single-enantiomer drugs with racemic precursors were based on RCTs directly comparing the 2 drugs.^15^

Introduction of the single-enantiomer drug is often timed to coincide with entry of generic competition of its racemic version,^5^ and many single-enantiomer drugs have become revenue blockbusters, shifting market share away from the generic versions of racemic predecessors.^18^ Previous systematic reviews have focused on specific medications and have suggested little to no efficacy or safety benefit with the new single-enantiomer formulations.^19,20,21^ In order for physicians and health care payers to understand the costs and benefits of these newer drugs, it is important to determine how often RCTs that directly compare single-enantiomer and racemic drugs are conducted and whether differences are observed for efficacy or safety end points. Accordingly, we systematically identified and evaluated the number, characteristics, and conclusions of RCTs that directly compared clinical efficacy or safety of single-enantiomer drugs to their racemic precursors.

## Methods

This systematic review was conducted from August 13, 2019, to January 7, 2021, using publicly available, nonclinical data. As a result, institutional review board approval or informed consent was not required. This study was conducted according to the Preferred Reporting Items for Systematic Reviews and Meta-analyses (PRISMA) reporting guideline.^22^ The study protocol was preregistered on PROSPERO.^23^

### Information Sources and Search

Three authors (A.S.L., A.D.Z., and A.C.E.) searched the publicly available Drugs@FDA database using the US Adopted Name prefixes for single-enantiomer drugs (ie, lev-/levo-, dex-/dextro-, ar-, and es-) to identify new single-enantiomer drug approvals up to March 1, 2019. As in prior work,^18^ we included New Drug Applications and excluded drugs that were duplicate, that were not part of a chiral pair of enantiomers based on the National Library of Medicine PubChem database, and that did not have an existing racemic formulation approved in the US or Europe. We also included drugs if they had an existing version that was a nonracemic mixture of enantiomers (eg, the precursor for dextroamphetamine is a 75:25 mixture of enantiomers). The manufacturer name and approval year for each identified drug and its racemic precursor were extracted from the Drugs@FDA database.

### Identification of RCTs Comparing Single-Enantiomer Drugs and Their Racemic Precursors

To identify all RCTs directly comparing a new single-enantiomer formulation of a drug with its previously approved racemic version (single-enantiomer racemic drug pairs), a systematic literature search was performed.^22^ In consultation with an experienced medical librarian (C.E.M.), we developed comprehensive search strategies using the generic and brand names for each drug, controlled vocabulary terms (where appropriate), and free-text words for each of the 15 pairs of drugs. The sensitivity-maximizing validated Cochrane filter was used to capture the RCT concept. Searches were conducted in Ovid MEDLINE (1946 to October 22, 2019), Ovid Embase (1974 to October 22, 2019), Web of Science Core Collection (all years), ClinicalTrials.gov, and Cochrane Central Registry of Controlled Trials (CENTRAL, Wiley, Issue 8 of 12, October 22, 2019) (eTable 1 in the Supplement).

### RCT Inclusion and Exclusion Criteria

We included all RCTs reported in peer-reviewed journal articles, conference abstracts, or trial registry databases that conducted direct statistical comparisons between single and racemic treatment arms for at least 1 efficacy and/or safety end point. We excluded studies that were not in English; that contained animals; that were observational, crossover, post hoc or pooled analyses; that were reviews, letters, or editorials; and/or that only evaluated pharmacodynamic or pharmacokinetic end points. Randomized clinical trials that only compared the single-enantiomer drugs or their racemic precursors against a placebo, nontherapeutic interventions, or other active drugs were excluded. We did not limit inclusion based on year, study population, or indication for drug use.

### Study Selection and Data Extraction

One investigator (A.S.L.) screened all identified articles at the title and abstract level. All potentially eligible studies were assessed in full text by 2 investigators (A.S.L. and J.D.W.), and uncertainties and discrepancies were resolved by consensus and discussion with 2 additional investigators (A.D.Z. and J.S.R). When multiple abstracts and full-text articles reported the same trial data, we prioritized full-text articles over published abstracts and results reported on clinical trial registries. For all eligible RCTs, we recorded whether industry support was reported, allocation (double-blind, single-blind, open-label, or unclear), intention-to-treat population for the direct comparison, study duration, ages included, sexes included (male, female, or both), indications, whether there were additional active treatment or placebo arms, and dose comparison (eg, single enantiomer at greater dose than racemic).

For each RCT, we first reviewed the Methods section and recorded any planned primary efficacy (ie, end points explicitly defined as primary in the Methods) and all safety end points. We then screened the Results section to identify all analyses directly comparing single-enantiomer and racemic drug arms and classified the corresponding end points as primary or secondary (ie, any other end point mentioned in the Methods section or reported in the Results section not explicitly defined as primary). Next, we classified each RCT as either (1) single enantiomer favored at the primary end point level, (2) racemic favored at the primary end point level, (3) single enantiomer favored at secondary end point level, (4) racemic favored at secondary end point level, or (5) neither drug favored. We considered all safety end points together and classified results as (1) single enantiomer favored, (2) neither drug favored, or (3) racemic favored. The classifications were based on whether the results favoring either drug were reported to achieve statistical significance based on each study’s defined significance level (eg, *P* < .05). Trials were characterized as favoring neither drug if no statistically significant differences were reported for any end point or if both drugs were favored for 1 or more end points. For efficacy, we first considered primary end points, and if no primary end point comparisons were reported or neither drug was favored, we then considered secondary end points. Efficacy and safety end points classifications were considered separately for each RCT. Therefore, a given RCT might favor one drug for efficacy and the other for safety.

Last, for all end points for which a statistically significant difference was reported between single-enantiomer racemic drug pairs, we abstracted whether the end point represented a clinical outcome (eg, mortality), a clinical scale (eg, Montgomery Asberg Depression Rating Scale), or a surrogate marker (eg, forced expiratory volume in 1 second).

### Statistical Analyses

We used descriptive statistics to summarize the study characteristics and findings of each RCT in our sample. We calculated proportions and medians (interquartile ranges). All analyses were conducted using MATLAB, version R2019a (MathWorks Inc).

## Results

### Characteristics of Single-Enantiomer and Racemic Drug Pairs

We identified 15 single-enantiomer drugs in the Drugs@FDA database with a preexisting racemic formulation approved in the US or Europe (Table 1). Although 14 of the drugs had racemic precursors available in the US, the precursor for eszopiclone, zopiclone, was only available outside of the US.

**Table 1.** Single-Enantiomer Drugs and Their Racemic Precursors^a^

Single-enantiomer drug (brand name)	Original manufacturer	Year approved	Racemic precursor (brand name)	Original manufacturer	Year approved
Arformoterol (Brovana)	Sepracor	2006	Formoterol (Foradil)	Novartis	2001
Armodafinil (Nuvigil)	Cephalon	2007	Modafinil (Provigil)	Cephalon	1998
Dexlansoprazole (Dexilant)	Takeda	2009	Lansoprazole (Prevacid)	Takeda	1995
Dexmethylphenidate (Focalin)	Novartis	2001	Methylphenidate (Ritalin)	Novartis	1955
Dextroamphetamine (Dexedrine)	Impax Labs	1976	Dextroamphetamine/amphetamine (Adderall)	Teva	1960
Escitalopram (Lexapro)	Forest Laboratories	2002	Citalopram (Celexa)	Forest Laboratories	1998
Esomeprazole (Nexium)	AstraZeneca	2001	Omeprazole (Prilosec)	AstraZeneca	1989
Eszopiclone (Lunesta)	Sepracor	2004	Zopiclone (Imovane/Zimovane)^b^	Rhone-Poulenc	1986
Levalbuterol (Xopenex)	Sepracor	1999	Albuterol (Proventil/Ventolin)	Schering/GlaxoSmithKline^c^	1981
Levobetaxolol (Betaxon)	Alcon	2000	Betaxolol (Betoptic)	Alcon	1985
Levobupivacaine (Chirocaine)	Purdue	1999	Bupivacaine (Marcaine)	Hospira	1972
Levocetirizine (Xyzal)	Sanofi-Aventis	2007	Cetirizine (Zyrtec)	Johnson and Johnson	1995
Levofloxacin (Levaquin)	Janssen	1996	Ofloxacin (Floxin)	Janssen	1980
Levoleucovorin (Fusilev)	Spectrum	2008	Leucovorin (Wellcovorin)	GlaxoSmithKline	1952
Levomilnacipran (Fetzima)	Allergan	2013	Milnacipran (Savella)	Allergan	2009

^a^ Approval information taken from Drugs@FDA database.

^b^ Approved in Europe.

^c^ Proventil manufactured by Schering; Ventolin manufactured by GlaxoSmithKline.

### Search Results

For the 15 single-enantiomer drugs with a preexisting racemic formulation, 15 041 articles and abstracts and 4081 records from ClinicalTrials.gov were identified through the literature search, of which 185 records reported on unique direct comparisons between single-enantiomer and racemic drug arms (eFigure and eTable 2 in the Supplement). One or more RCTs were identified for levobupivacaine (124 [67.0%]), arformoterol (1 [0.5%]), armodafinil (1 [0.5%]), dexmethylphenidate (1 [0.5%]), eszopiclone (1 [0.5%]), levoleucovorin (2 [1.1%]), levocetirizine (3 [1.6%]), dexlansoprazole (4 [2.2%]), levofloxacin (4 [2.2%]), escitalopram (8 [4.3%]), esomeprazole (17 [9.2%]), and levalbuterol (19 [10.3%]) (Table 2). No eligible RCTs were identified for dextroamphetamine, levobetaxolol, and levomilnacipran. The median number of RCTs identified for each of the 15 pairs of drugs was 2 (interquartile range [IQR], 1-8).

**Table 2.** Characteristics of Randomized Clinical Trials (RCTs) Directly Comparing Single-Enantiomer Drugs to Their Racemic Precursors

RCT characteristic	No. (%)												
	Arformoterol vs Formoterol	Armodafinil vs Modafinil	Dexlansoprazole vs Lansoprazole	Dexmethylphenidate vs Methylphenidate	Escitalopram vs Citalopram	Esomeprazole vs Omeprazole	Eszopiclone vs Zopiclone	Levalbuterol vs Albuterol	Levobupivacaine vs Bupivacaine	Levocetirizine vs Cetirizine	Levofloxacin vs Ofloxacin	Levoleucovorin vs Leucovorin	All
**No. of RCTs**	**1**	**1**	**4**^**a**^	**1**	**8**	**17**^**a**^	**1**	**19**	**124**	**3**	**4**	**2**	**185**
Article type													
Abstract only^b^	0	0	1 (25)	0	1 (13)	2 (12)	0	2 (11)	19 (15.3)	2 (33)	1 (25)	0	28 (15.1)
Full text	1 (100)	1 (100)	3 (75)	1 (100)	7 (88)	15 (88)	1 (100)	17 (89)	105 (84.7)	1 (67)	3 (75)	2 (100)	157 (84.9)
Industry funding or author affiliations													
Any industry	1 (100)	1 (100)	3 (75)	1 (100)	6 (75)	11 (65)	1 (100)	11 (58)	17 (13.7)	1 (33)	3 (75)	0	56 (30.3)
Only nonindustry	0	0	1 (25)	0	2 (25)	3 (18)	0 (0)	4 (21)	30 (24.2)	0	0	1 (50)	41 (22.2)
Unclear	0	0	0	0	0	3 (18)	0 (0)	4 (21)	77 (62.1)	2 (67)	1 (25)	1 (50)	88 (47.6)
Age^c^													
Pediatric only	0	0	0	1 (100)	0	0	0	8 (42)	10 (8.1)	1 (33)	1 (25)	0	21 (11.4)
Adult/elderly only	1 (100)	1 (100)	4 (100)	0	7 (87)	15 (88)	1 (100)	6 (32)	103 (83.1)	0	1 (25)	2 (100)	141 (76.2)
Pediatric and adult/elderly	0	0	0	0	0	0	0	4 (21)	4 (3.2)	1 (33)	2 (50)	0	11 (5.9)
Unclear	0	0	0	0	1 (13)	2 (12)	0	1 (5)	7 (5.6)	1 (33)	0	0	12 (6.5)
Sex													
Male only	0	0	0	0	1 (13)	0	0	0	12 (9.7)	0	0	0	13 (7.0)
Female only	0	0	0	0	0	0	0	0	36 (29.0)	0	1 (25)	0	37 (20.0)
Both	1 (100)	1 (100)	4 (100)	1 (100)	7 (88)	16 (94)	1 (100)	18 (95)	64 (51.6)	2 (67)	2 (50)	2 (100)	119 (64.3)
Unclear	0	0	0	0	0	1 (6)	0	1 (5)	12 (9.7)	1 (33)	1 (25)	0 (0)	16 (8.6)
Enrollment, median (IQR)	444 (444-444)	211 (211-211)	1149 (245.5-2042)	90 (90-90)	322.5 (280.5-358)	448 (156-1148)	199 (199-199)	128 (80.5-280)	60 (50-80)	60 (55-263)	234.5 (46-465.8)	564 (383-745)	70 (56-150)
Patient follow-up, median (IQR), d	182 (182-182)	84 (84-84)	56 (56-56)	28 (28-28)	56 (42-56)	56 (28-56)	28 (28-28)	28 (1-30)	1 (1-1)	28 (21-56)	10 (9.3-18)	2007.5 (2007.5-2007.5)	1 (1-28)
Allocation													
Double-blind	1 (100)	1 (100)	3 (75)	1 (100)	8 (100)	11 (65)	1 (100)	13 (68)	98 (79.0)	2 (67)	3 (75)	0	142 (76.8)
Single-blind	0	0	0	0	0	2 (12)	0	0	14 (11.3)	0	0	0	16 (8.6)
Open-label	0	0	1 (25)	0	0	1 (6)	0	4 (21)	1 (0.8)	1 (33)	0	0	8 (4.3)
Unclear	0	0	0	0	0	3 (18)	0	2 (11)	11 (8.9)	0	1 (25)	2 (100)	19 (10.3)
Treatment arms													
Single enantiomer and racemic only	1 (100)	1 (100)	4 (100)	0	6 (75)	16 (94)	1 (100)	13 (68)	97 (78.2)	2 (67)	4 (100)	2 (100)	147 (79.5)
Including other arms	0	0	0	1 (100)	2 (25)	1 (6)	0	6 (32)	27 (21.8)	1 (33)	0	0	38 (20.5)
Dose comparisons													
Single enantiomer at higher dose	1 (100)	0	4 (100)	0	0	9 (53)	0	0	1 (0.8)	0	3 (75)	0	18 (9.7)
Single enantiomer and racemic at the same dose	0	0	0	0	0	6 (35)	0	0	111 (89.5)	0	0	1 (50)	118 (63.8)
Single enantiomer at a lower dose	0	1 (100)	0	1 (100)	7 (88)	0	1 (100)	18 (95)	1 (0.8)	3 (100)	1 (25)	1 (50)	34 (18.4)
Multiple or unclear dose comparisons	0	0	0	0	1 (12)	2 (12)	0	1 (5)	11 (8.9)	0	0	0	15 (8.1)
Primary end points													
Prespecified in Methods	1 (100)	1 (100)	4 (100)	1 (100)	7 (88)	15 (88)	1 (100)	15 (79)	44 (35.5)	1 (33)	1 (25)	1 (50)	92 (49.7)
Safety analysis													
Safety prespecified in Methods	1 (100)	1 (100)	2 (50)	1 (100)	7 (88)	11 (65)	1 (100)	15 (79)	98 (79.0)	2 (67)	3 (75)	2 (100)	144 (77.8)

Abbreviation: IQR, interquartile range.

^a^ For 2 dexlansoprazole RCTs and 3 esomeprazole RCTs, the results were published as part of a pooled analysis. Comparisons were abstracted separately for efficacy and pooled for safety.

^b^ Including unpublished trials with results available from government registries.

^c^ Pediatric, <18 y; adult, 18-65 y; elderly, >65 y.

### General Characteristics of Included RCTs

Of the 185 eligible RCTs reporting on direct comparisons between single-enantiomer drugs and their racemic precursors, 157 (84.9%) were full-text articles, and 28 (15.1%) were published abstracts or unpublished results obtained from a clinical trial registry (Table 2). The majority of RCTs were double-blind (142 [76.8%]), were focused on adult and/or elderly patients (141 [76.2%]), and included both men and women (119 [64.3%]). Median trial enrollment was 70 patients (IQR, 56-150 patients), and median length of follow-up was 1 day (IQR, 1-28 days). Excluding the 124 levobupivacaine RCTs (67.0%), median trial enrollment was 260 patients (IQR, 100-547 patients), and median length of follow-up was 42 days (IQR, 23-56 days). There were 56 RCTs (30.2%) that disclosed industry funding or had authors who disclosed industry affiliations.

Of the 185 RCTs making at least 1 direct comparison between single-enantiomer and racemic drug arms for any efficacy and/or safety end point, 179 (96.8%) and 124 (67.0%) made at least 1 comparison for an efficacy or safety end point, respectively. Two-thirds of the RCTs (118 [63.8%]) compared the single-enantiomer and racemic drugs at the same dose. There were 18 (9.7%) RCTs with the single enantiomer at a higher dose, 34 (18.4%) with the racemic at a higher dose, and 15 (8.1%) with multiple or unclear dose comparisons (Table 2).

### Efficacy Comparisons

**Figure 1.**
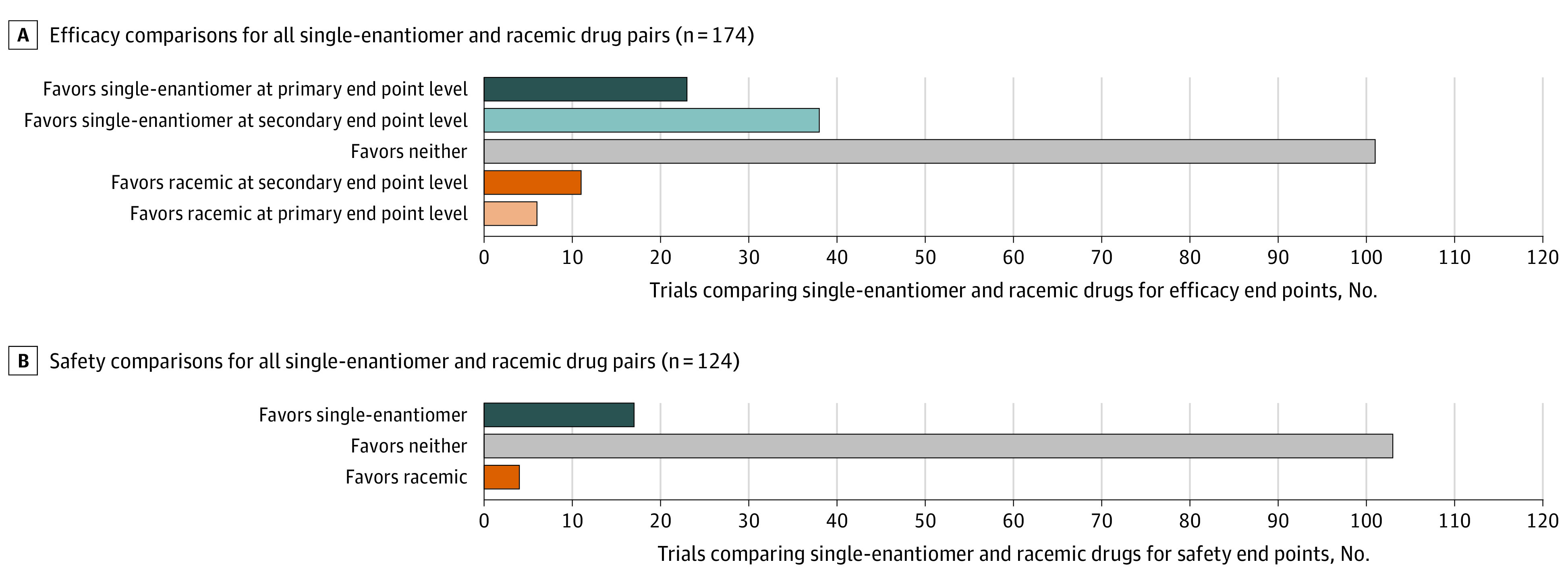
Combined Findings of All Randomized Clinical Trials Directly Comparing Single-Enantiomer and Racemic Drug Pairs Comparisons of efficacy (A) and safety (B) for all single-enantiomer and racemic drug pairs.

**Figure 2.**
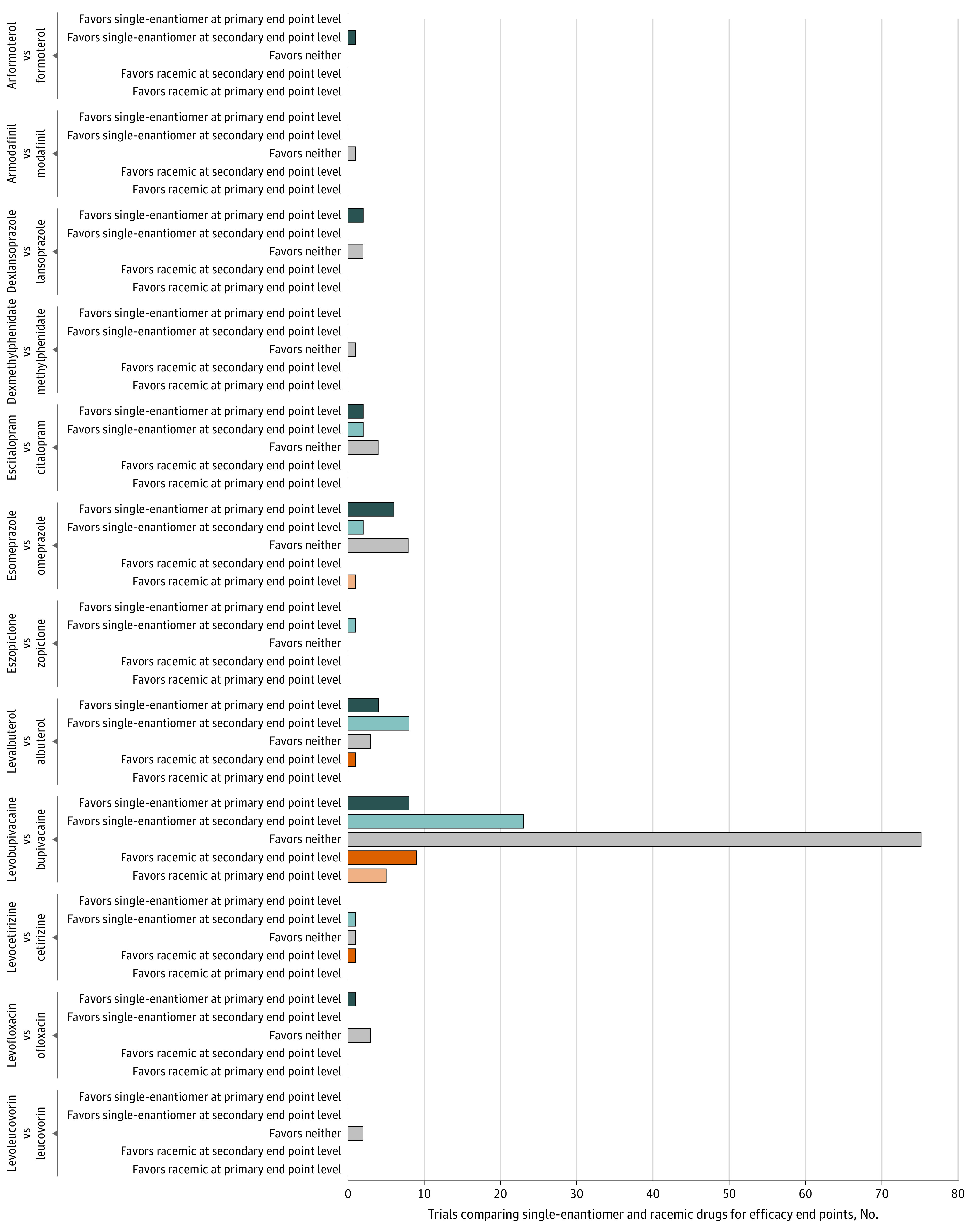
Findings by Drug Pair of Randomized Clinical Trials Directly Comparing Single-Enantiomer and Racemic Drug Pairs for Efficacy End Points

There were 174 RCTs directly comparing drug pairs using efficacy end points. A total of 23 RCTs (12.8%) favored the single enantiomer based on a primary end point, and 6 (3.4%) favored the racemic drug based on a primary end point. There were 38 RCTs (21.2%) that favored the single enantiomer based on a secondary end point, 11 (6.1%) that favored the racemic based on a secondary end point, and 101 (56.4%) that favored neither drug (Figure 1, Figure 2, and eTable 3 in the Supplement). Among the 15 drug pairs, we identified at least 1 RCT offering evidence of primary efficacy end point benefit for 6 single-enantiomer drugs and 3 racemic drugs.

For the 23 RCTs favoring a single-enantiomer drug and 6 RCTs favoring a racemic drug based on the primary end points, 12 of 23 (52%) and 5 of 6 (83%) of these primary end points were clinical scales or clinical outcomes, whereas 11 of 23 (48%) and 1 of 6 (17%) were surrogate markers (eTable 4 in the Supplement). For the 38 RCTs favoring a single-enantiomer drug and 11 RCTs favoring a racemic drug based on secondary end points, 31 of 38 (82%) and 9 of 11 (82%) were clinical scales or clinical outcomes, whereas 7 of 38 (18%) and 2 of 11 (18%) were surrogate markers (eTable 4 in the Supplement).

### Safety Comparisons

**Figure 3.**
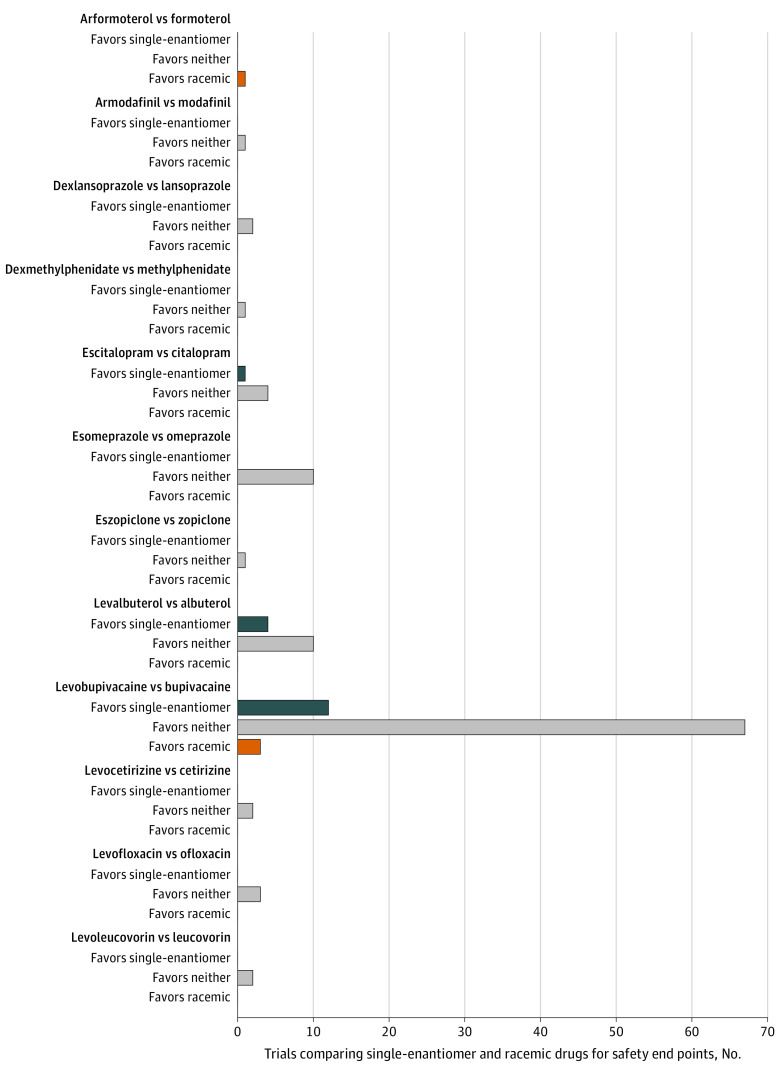
Findings by Drug Pair of Randomized Clinical Trials Directly Comparing Single-Enantiomer and Racemic Drug Pairs for Safety End Points

There were 124 RCTs directly comparing drug pairs using safety end points, of which 5 (4.0%) were primary safety end points. Seventeen of 124 (13.7%) of the safety comparisons favored the single enantiomer, 4 (3.2%) favored the racemic drug, and 103 (83.1%) favored neither drug (Figure 1 and Figure 3). Among the 15 drug pairs, we identified at least 1 RCT offering evidence of safety benefit for 3 single-enantiomer drugs and 2 racemic drugs (Figure 3). Safety differences were most often based on vital sign measurements (eg, blood pressure), laboratory tests (eg, serum potassium), or frequency of all or specific adverse events (eTable 5 in the Supplement).

## Discussion

We systematically identified and evaluated the number, characteristics, and outcomes of RCTs directly comparing clinical efficacy or safety of all FDA-approved single-enantiomer drugs with their racemic precursors. We found that for many newly marketed single-enantiomer drugs, few RCTs were conducted that made direct comparisons with their racemic precursors for efficacy or safety end points. When single-enantiomer drugs were directly compared with their racemic precursors, the majority of RCTs favored neither drug on the basis of efficacy or safety, and RCTs tended to be small and without well-defined end points. For only 6 of the 15 single-enantiomer drugs was there at least 1 RCT that demonstrated improved efficacy based on a primary end point when compared with its racemic precursor. For only 3 of these 6 single-enantiomer drugs was there at least 1 RCT that demonstrated improved safety. Thus, for 9 of the 15 FDA-approved single-enantiomer drugs, our results suggest that there was no RCT evidence of improved efficacy, based on primary end point results, or safety as compared with their racemic precursors. Our findings highlight the need for physicians and payers to critically assess whether the higher costs to patients and the health care system associated with these newer drugs are justified.

Single-enantiomer drugs were rarely found to be superior to their racemic precursors based on the RCTs’ primary efficacy end point results. Although RCTs were identified favoring single-enantiomer drugs based on nonpredefined and secondary end points, results from predefined primary end points should take priority over other analyses.^24^ Randomized clinical trials are typically powered to assess primary end points, and secondary analyses may be more susceptible to false positive findings.^24^ Furthermore, many of the RCTs had small sample sizes and compared the drugs at noncomparable doses, making them less suitable for determining evidence of clinical superiority. Concerns have been raised that some of the apparent benefits shown by single-enantiomer drugs may be due to RCTs using noncomparable doses of the single-enantiomer and racemic drugs.^15^ For 3 drugs in our sample—esomeprazole, dexlansoprazole, and levofloxacin—the vast majority of the RCTs evaluated the single enantiomer at a higher dose (ie, at least twice the dose of the active enantiomer as their racemic precursors), making it difficult to determine whether these newer drugs provide benefits at therapeutically similar doses.

Our study identified RCTs favoring a single-enantiomer drug over its racemic precursor based on any safety end point for only 3 of 15 drugs, and differences were often observed for a single adverse event or vital sign without clear clinical relevance. Benefits claimed with chiral switching often revolve around safety,^5,14^ and previous reviews relying on preclinical evidence or trials in healthy volunteers have suggested that single-enantiomer drugs are safer than racemic drugs.^12,25,26^ For example, according to animal studies and trials in healthy volunteers, levobupivacaine, which accounted for two-thirds of identified RCTs, was believed to pose less risk of cardiac and central nervous system toxicity than its precursor, bupivacaine.^11^ A large number of RCTs have been conducted comparing levobupivacaine and bupivacaine, likely because these drugs are used in a wide variety of procedures for only a short duration. Levobupivacaine was only briefly marketed in the US owing to antitrust concerns,^27^ but it was first approved in Sweden and has been used more widely in Europe. In our study, levobupivacaine was favored over bupivacaine based on any safety measure in only one-fifth of RCTs identified. For the approval of single-enantiomers, the FDA requires only minimal comparative studies focused on demonstrating that the single enantiomer is not more dangerous, and manufacturers can use safety data previously collected on the racemic precursors. Our results suggest that claims of superior safety of single-enantiomer drugs compared with their racemic precursor have not borne out convincingly in RCTs conducted before or after FDA approval.

Our findings highlight potentially wasteful spending on expensive brand-name single-enantiomer drugs without proven benefit over already marketed racemic drugs. Recent estimates suggest that Medicare could have saved $17.7 billion between 2011 and 2017 if spending on single-enantiomer drugs was substituted with their generic racemic precursors.^18^ Spending estimates outside of the US are lacking, but many single-enantiomer drugs are first approved in or marketed exclusively in Europe, where their racemic precursors are also in use.^5^ Given the lack of direct-comparison RCTs informing FDA approval of single-enantiomer versions of racemic drugs,^15^ payers should demand rigorous studies supporting claims of efficacy and safety benefits of single-enantiomers before covering them over generic racemic precursors. If direct-comparison RCTs are not considered, single-enantiomer versions of racemic drugs will continue to be rapidly integrated into standard treatment based largely on preclinical evidence, and postapproval studies are likely to be disincentivized by the risk of moderating previous perceptions of efficacy and safety.^28^

### Limitations

Our study has several limitations. First, we did not conduct any meta-analyses to determine the magnitude of differences for specific comparisons, indications, and end points. Meta-analyses would not have been feasible given the heterogeneity of indications and end points reported across the included RCTs. Second, risk of bias assessments were not conducted to quantify the quality of the studies and/or stratify our results across studies with high or low risk of bias. Our objective was to provide an overview of all RCTs directly comparing single-enantiomer racemic drug pairs for efficacy or safety end points, including specific features associated with quality. Third, we limited our study to English-language RCTs reporting direct statistical comparisons between single-enantiomer racemic drug pairs. However, it is unlikely that studies not conducting direct comparisons would change our overall findings. Lastly, we did not attempt to identify and summarize findings reported in observational studies, which could provide insight regarding the real-world comparative efficacy and safety of single-enantiomer and racemic drugs.

## Conclusions

The results of this systematic review suggest that newly marketed, FDA-approved single-enantiomer drugs are infrequently directly compared with racemic precursors, and when they are, they are uncommonly found to provide improved efficacy or safety. These findings raise concerns about the greater costs to the health care system incurred by chiral switching, without evidence to support benefit to patients, and the need for physicians and payers to encourage or require high-quality direct-comparison RCTs to inform the use of these more expensive therapeutics.

## Notes

Supplement. **eFigure.** Study Flow Chart **eTable 1.** Medline Search Strategy **eTable 2.** Characteristics of Included Studies **eTable 3.** Results of Efficacy and Safety Comparisons **eTable 4.** Efficacy End Point Classification for Randomized Clinical Trials Favoring Single-Enantiomer or Racemic Drugs **eTable 5.** Descriptions of Safety End Points for Randomized Clinical Trials Favoring Single-Enantiomer or Racemic Drugs
