# Supplementary material for: Evaluation of Trials Comparing Single-Enantiomer Drugs to Their Racemic Precursors: A Systematic Review
Source: JAMA Netw Open. 2021 May 6;4(5):e215731. doi: 10.1001/jamanetworkopen.2021.5731 (PMC8103227; doi:10.1001/jamanetworkopen.2021.5731)
Supplement: Supplement. — eFigure. Study Flow Chart eTable 1. Medline Search Strategy eTable 2. Characteristics of Included Studies eTable 3. Results of Efficacy and Safety Comparisons eTable 4. Efficacy End Point Classification for Randomized Clinical Trials Favoring Single-Enantiomer or Racemic Drugs eTable 5. Descriptions of Safety End Points for Randomized Clinical Trials Favoring Single-Enantiomer or Racemic Drugs [file jamanetwopen-e215731-s001.pdf]

Supplementary Online Content

Long AS, Zhang AD, Meyer CE, Egilman AC, Ross JS, Wallach JD. Evaluation of trials comparing single-enantiomer drugs to their racemic precursors: a systematic review. *JAMA Netw Open*. 2021;4(5):e215731. doi:10.1001/jamanetworkopen.2021.5731

eFigure. Study Flow Chart

eTable 1. Medline Search Strategy

eTable 2. Characteristics of Included Studies

eTable 3. Results of Efficacy and Safety Comparisons

eTable 4. Efficacy End Point Classification for Randomized Clinical Trials Favoring Single-Enantiomer or Racemic Drugs

eTable 5. Descriptions of Safety End Points for Randomized Clinical Trials Favoring Single-Enantiomer or Racemic Drugs

This supplementary material has been provided by the authors to give readers additional information about their work.

© 2021 Long AS et al. *JAMA Network Open.*

Figure. Study Flow Chart

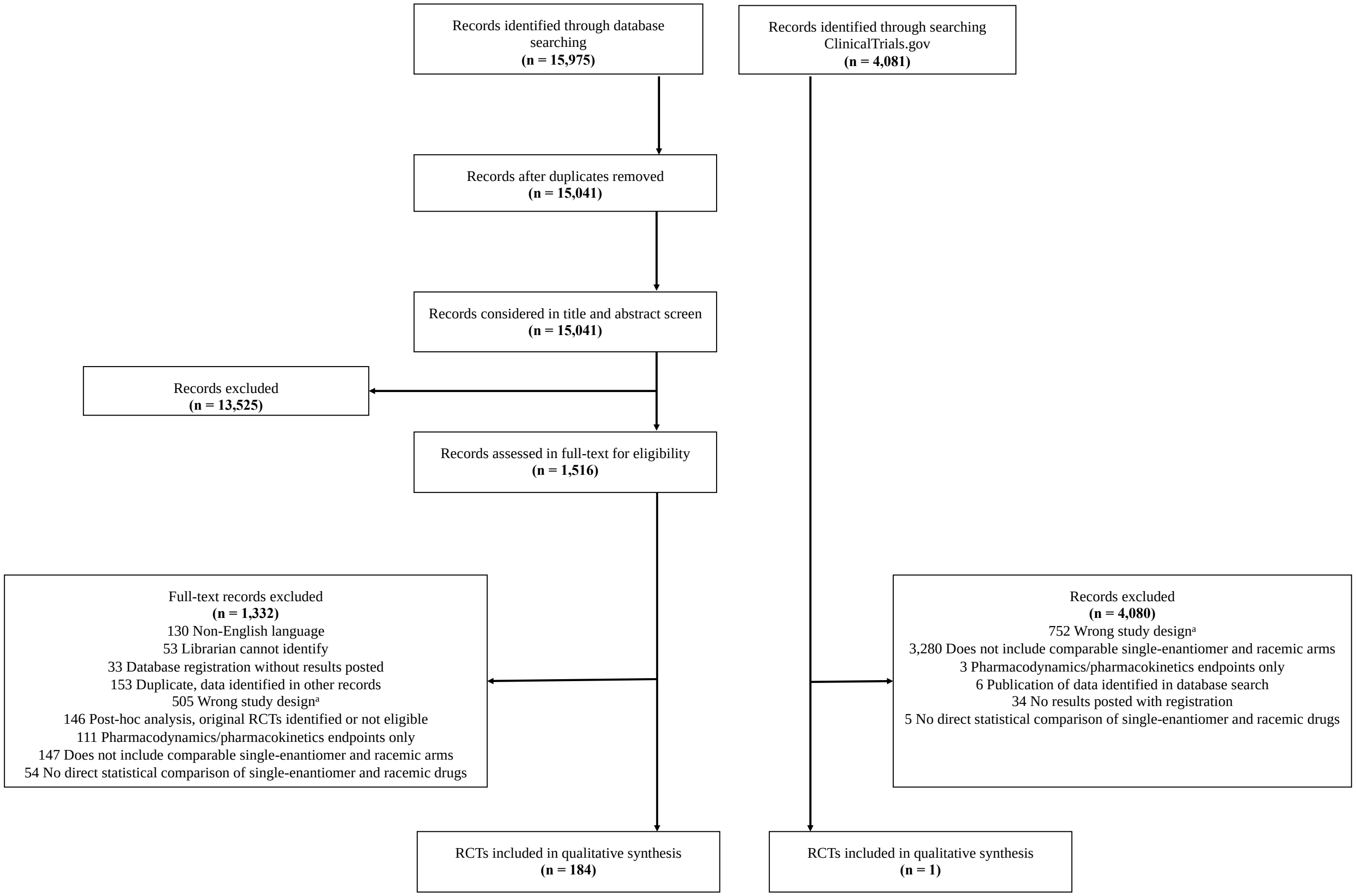

a) Wrong study design includes Non-Clinical Research, Reviews, Editorials, Letters, Observational Design, Crossover Design, Non-randomized

© 2021 Long AS et al. *JAMA Network Open.*

**eTable1.** Medline Search Strategy[a]	
**Arformoterol vs. Formoterol**	1. [arformoterol vs formoterol]<br>2. arformoterol.mp.<br>3. arformoterol tartrate.mp.<br>4. brovana.mp.<br>5. 2 or 3 or 4<br>6. [deleted MeSH term because both drugs were indexed with it]<br>7. formoterol.mp.<br>8. formoterol fumarate.mp.<br>9. perforomist.mp.<br>10. foradil.mp.<br>11. foradil certihaler.mp.<br>12. 6 or 7 or 8 or 9 or 10 or 11<br>13. [clinical trials]<br>14. randomized controlled trial.pt.<br>15. controlled clinical trial.pt.<br>16. randomized.ab.<br>17. placebo.ab.<br>18. drug therapy.fs.<br>19. randomly.ab.<br>20. trial.ab.<br>21. groups.ab.<br>22. 14 or 15 or 16 or 17 or 18 or 19 or 20 or 21<br>23. exp animals/ not humans.sh.<br>24. 22 not 23<br>25. [ANDing]<br>26. 5 and 12 and 24<br>27. "(R,R)-formoterol".mp.<br>28. r-formoterol.mp.<br>29. 5 or 27 or 28<br>30. 12 and 24 and 29
**Armodafinil vs. Modafinil**	1. ("(R)-Modafinil" or "(R)-(-)-Modafinil" or "l-Modafinil" or "(-)-(R)-modafinil" or "r-modafinil").mp.<br>2. [armodafinil vs modafinil]<br>3. armodafinil.mp.<br>4. nuvigil.mp.<br>5. 3 or 4<br>6. [MeSH term deleted because term is used for both drugs]<br>7. modafinil.mp.<br>8. provigil.mp.<br>9. 6 or 7 or 8<br>10. [clinical trials]<br>11. randomized controlled trial.pt.

© 2021 Long AS et al. *JAMA Network Open.*

	12. controlled clinical trial.pt.<br>13. randomized.ab.<br>14. placebo.ab.<br>15. drug therapy.fs.<br>16. randomly.ab.<br>17. trial.ab.<br>18. groups.ab.<br>19. 11 or 12 or 13 or 14 or 15 or 16 or 17 or 18<br>20. exp animals/ not humans.sh.<br>21. 19 not 20<br>22. [anding together]<br>23. 5 and 9 and 21<br>24. 1 or 3 or 4<br>25. 9 and 21 and 24
**Dexlansoprazole vs. Lansoprazole**	1. [dexlansoprazole vs lansoprazole]<br>2. exp Dexlansoprazole/<br>3. dexlansoprazole.mp.<br>4. dexilant.mp.<br>5. dexilant solutab.mp.<br>6. 2 or 3 or 4 or 5<br>7. Lansoprazole/<br>8. lansoprazole.mp.<br>9. prevacid.mp.<br>10. prevacid iv.mp.<br>11. 7 or 8 or 9 or 10<br>12. [clinical trials]<br>13. randomized controlled trial.pt.<br>14. controlled clinical trial.pt.<br>15. randomized.ab.<br>16. placebo.ab.<br>17. drug therapy.fs.<br>18. randomly.ab.<br>19. trial.ab.<br>20. groups.ab.<br>21. 13 or 14 or 15 or 16 or 17 or 18 or 19 or 20<br>22. exp animals/ not humans.sh.<br>23. 21 not 22<br>24. [anding together]<br>25. 6 and 11 and 23<br>26. ("(R)-Lansoprazole" or "R-(+)-LANSOPRAZOLE" or "(r)-(+)-lansoprazole" or "(+)-(R)-Lansoprazole" or "(+)-lansoprazol" or "d-lansoprazole").mp.<br>27. 6 or 26<br>28. 11 and 23 and 27

© 2021 Long AS et al. *JAMA Network Open.*

**Dexmethylphenidate vs. Methylphenidate**	1. (D-TMP or d-threo-Methylphenidate or d-Methylphenidate or "(+)-threo-Methylphenidate" or "Methyl D-phenidate" or "Dex methylphenidate" or "D-MPH" or "threo-(+)-Methylphenidate" or "dex-methylphenidate" or "d-threo methylphenidate" or dextromethylphenidate).mp.<br>2. [dexmethylphenidate vs methylphenidate]<br>3. exp Dexmethylphenidate Hydrochloride/<br>4. Dexmethylphenidate Hydrochloride.mp.<br>5. Dexmethylphenidate.mp.<br>6. focalin.mp. [mp=title, abstract, original title, name of substance word, subject heading word, floating sub-heading word, keyword heading word, organism supplementary concept word, protocol supplementary concept word, rare disease supplementary concept word, unique identifier, synonyms]<br>7. focalin xr.mp.<br>8. 3 or 4 or 5 or 6 or 7<br>9. Methylphenidate/<br>10. Methylphenidate.mp.<br>11. Methylphenidate hydrochloride.mp.<br>12. ritalin.mp.<br>13. ritalin LA.mp.<br>14. ritalin-sr.mp.<br>15. quillivant xr.mp.<br>16. quillichew er.mp.<br>17. methylin.mp.<br>18. metadate cd.mp.<br>19. metadate er.mp.<br>20. jornay pm.mp.<br>21. daytrana.mp.<br>22. cotempla xr-odt.mp.<br>23. concerta.mp.<br>24. aptensio xr.mp.<br>25. adhansia xr.mp.<br>26. or/9-25<br>27. [clinical trials]<br>28. randomized controlled trial.pt.<br>29. controlled clinical trial.pt.<br>30. randomized.ab.<br>31. placebo.ab.<br>32. drug therapy.fs.<br>33. randomly.ab.<br>34. trial.ab.<br>35. groups.ab.<br>36. 28 or 29 or 30 or 31 or 32 or 33 or 34 or 35<br>37. exp animals/ not humans.sh.<br>38. 36 not 37<br>39. [ANDing together drugs]<br>40. 8 and 26 and 38<br>41. 1 or 8

© 2021 Long AS et al. *JAMA Network Open.*

	42. 26 and 38 and 41
**Dextroampetimine vs Amphetamine**	1. [dextroamphetamine vs amphetamine]<br>2. exp Dextroamphetamine/<br>3. dextroamphetamine.mp.<br>4. dextroamphetamine sulfate.mp.<br>5. dexedrine.mp.<br>6. dextrostat.mp.<br>7. ferndex.mp.<br>8. dexampex.mp.<br>9. 2 or 3 or 4 or 5 or 6 or 7 or 8<br>10. Amphetamine/<br>11. amphetamine.mp.<br>12. mixed amphetamine salt.mp.<br>13. adderall.mp.<br>14. adderall xr.mp.<br>15. mydayis.mp.<br>16. dextroamp.mp.<br>17. 10 or 11 or 12 or 13 or 14 or 15 or 16<br>18. [clinical trials]<br>19. randomized controlled trial.pt.<br>20. controlled clinical trial.pt.<br>21. randomized.ab.<br>22. placebo.ab.<br>23. drug therapy.fs.<br>24. randomly.ab.<br>25. trial.ab.<br>26. groups.ab.<br>27. 19 or 20 or 21 or 22 or 23 or 24 or 25 or 26<br>28. exp animals/ not humans.sh.<br>29. 27 not 28<br>30. [ANDing]<br>31. 9 and 17 and 29<br>32. (Dexamfetamine or "dextro Amphetamine" or "Dexedrine Spansule" or Dexadrine or "(S)-(+)-Amphetamine" or "D-Amphetamine" or "Dexamphetamine" or "Dexamfetamine" or "(S)-Amphetamine" or "(+)-(S)-Amphetamine" or "D-(S)-Amphetamine" or "(2S)-(+)-Amphetamine" or "dextro-Amphetamine" or "D-(+)-Amphetamine" or "S(+)-Amphetamine" or "S(+)amphetamine").mp.<br>33. 32 or 9<br>34. 33 or 17 or 29
**Escitalopram vs. Citalopram**	1. [escitalopram vs citalopram]<br>2. escitalopram.mp.<br>3. escitalopram oxolate.mp.<br>4. lexapro.mp.<br>5. 2 or 3 or 4<br>6. [removed MeSH term citalopram because both drugs were indexed with it]<br>7. citalopram.mp.

© 2021 Long AS et al. *JAMA Network Open.*

	8. citalopram hydrobromide.mp.<br>9. celexa.mp.<br>10. 6 or 7 or 8 or 9<br>11. [clinical trials]<br>12. randomized controlled trial.pt.<br>13. controlled clinical trial.pt.<br>14. randomized.ab.<br>15. placebo.ab.<br>16. drug therapy.fs.<br>17. randomly.ab.<br>18. trial.ab.<br>19. groups.ab.<br>20. 12 or 13 or 14 or 15 or 16 or 17 or 18 or 19<br>21. exp animals/ not humans.sh.<br>22. 20 not 21<br>23. [ANDing together]<br>24. 5 and 10 and 22<br>25. ("(S)-Citalopram" or "S(+)-Citalopram" or "S-(+)-Citalopram" or "(S)-(+)-Citalopram Oxalate").mp.<br>26. 5 or 25<br>27. 10 and 22 and 26
**Esomeprazole vs. Omeprazole**	1. [esomeprazole vs omeprazole]<br>2. exp Esomeprazole/<br>3. esomeprazole.mp.<br>4. esomeprazole magnesium.mp.<br>5. esomeprazole strontium.mp.<br>6. nexium.mp.<br>7. nexium 24HR.mp.<br>8. nexium iv.mp.<br>9. 2 or 3 or 4 or 5 or 6 or 7 or 8<br>10. Omeprazole/<br>11. omeprazole.mp.<br>12. omeprazole magnesium.mp.<br>13. prilosec.mp.<br>14. prilosec OTC.mp.<br>15. 10 or 11 or 12 or 13 or 14<br>16. [clinical trials]<br>17. randomized controlled trial.pt.<br>18. controlled clinical trial.pt.<br>19. randomized.ab.<br>20. placebo.ab.<br>21. drug therapy.fs.<br>22. randomly.ab.<br>23. trial.ab.<br>24. groups.ab.

© 2021 Long AS et al. *JAMA Network Open.*

	25. 17 or 18 or 19 or 20 or 21 or 22 or 23 or 24<br>26. exp animals/ not humans.sh.<br>27. 25 not 26<br>28. [ANDing it all together]<br>29. 9 and 15 and 27<br>30. ("esomeprazole sodium OR esomeprazol" or "(S)-Omeprazole" or "(S)-(-)-Omeprazole" or "s-omeprazole").mp.<br>31. 30 or 9<br>32. 31 and 15 and 27
**Eszopiclone vs. Zopiclone**	1. [eszopiclone vs zopiclone]<br>2. exp Eszopiclone/<br>3. eszopiclone.mp.<br>4. lunesta.mp.<br>5. 2 or 3 or 4<br>6. zopiclone.mp.<br>7. [clinical trials]<br>8. randomized controlled trial.pt.<br>9. controlled clinical trial.pt.<br>10. randomized.ab.<br>11. placebo.ab.<br>12. drug therapy.fs.<br>13. randomly.ab.<br>14. trial.ab.<br>15. groups.ab.<br>16. 8 or 9 or 10 or 11 or 12 or 13 or 14 or 15<br>17. exp animals/ not humans.sh.<br>18. 16 not 17<br>19. [ANDing]<br>20. 5 and 6 and 18<br>21. ("(S)-Zopiclone" or "(S)-Eszopiclone" or "(S)-(+)-Zopiclone" or s-zopiclone).mp.<br>22. 5 or 21<br>23. (Imovane or Zimovane or Amovane).mp.<br>24. 6 or 23<br>25. 18 and 22 and 24
**Levalbuterol vs. Albuterol**	1. [levalbuterol/levosalbutamol vs albuterol/salbutamol]<br>2. exp Levalbuterol/<br>3. levalbuterol.mp.<br>4. levosalbutamol.mp.<br>5. levalbuterol hydrochloride.mp.<br>6. xopenex.mp.<br>7. xopenex hfa.mp.<br>8. 2 or 3 or 4 or 5 or 6 or 7<br>9. Albuterol/<br>10. albuterol.mp.

© 2021 Long AS et al. *JAMA Network Open.*

	11. salbutamol.mp.<br>12. albuterol sulfate.mp.<br>13. proventil.mp.<br>14. proventil hfa.mp.<br>15. ventolin.mp.<br>16. ventolin hfa.mp.<br>17. ventolin rotacaps.mp.<br>18. accuneb.mp.<br>19. proair hfa.mp.<br>20. proair digihaler.mp.<br>21. volmax.mp.<br>22. vospire er.mp.<br>23. or/9-22<br>24. [clinical trials]<br>25. randomized controlled trial.pt.<br>26. controlled clinical trial.pt.<br>27. randomized.ab.<br>28. placebo.ab.<br>29. drug therapy.fs.<br>30. randomly.ab.<br>31. trial.ab.<br>32. groups.ab.<br>33. 25 or 26 or 27 or 28 or 29 or 30 or 31 or 32<br>34. exp animals/ not humans.sh.<br>35. 33 not 34<br>36. [anding together]<br>37. 8 and 23 and 35<br>38. ("(R)-salbutamol" or "R-Salbutamol" or "R-Albuterol" or "(r)-(-)-salbutamol" or "(R)-albuterol" or "(R) - Albuterol" or "(R)-(-)-Albuterol").mp.<br>39. 8 or 38<br>40. 39 and 23 and 35
**Levobetaxolol vs. Betaxolol**	1. [levobetaxolol vs betaxolol]<br>2. levobetaxolol.mp.<br>3. betaxon.mp.<br>4. 2 or 3<br>5. [removing MeSH term because it's used for both drugs]<br>6. betaxolol.mp.<br>7. betaxolol hydrochloride.mp.<br>8. betoptic.mp.<br>9. kerlone.mp.<br>10. 5 or 6 or 7 or 8 or 9<br>11. [clinical trials]<br>12. randomized controlled trial.pt.<br>13. controlled clinical trial.pt.<br>14. randomized.ab.

© 2021 Long AS et al. *JAMA Network Open.*

	15. placebo.ab.<br>16. drug therapy.fs.<br>17. randomly.ab.<br>18. trial.ab.<br>19. groups.ab.<br>20. 12 or 13 or 14 or 15 or 16 or 17 or 18 or 19<br>21. exp animals/ not humans.sh.<br>22. 20 not 21<br>23. [ANDing]<br>24. 4 and 10 and 22<br>25. ("(S)-Betaxolol" or "(S)-(-)-Betaxolol" or "Betaxon" or "l-betaxolol").mp.<br>26. 4 or 25<br>27. 10 and 22 and 26
**Levobupivacaine vs. Bupivacaine**	1. [levobupivacaine vs bupivacaine]<br>2. exp Levobupivacaine/<br>3. levobupivacaine.mp.<br>4. chirocaine.mp.<br>5. 2 or 3 or 4<br>6. exp Bupivacaine/<br>7. bupivacaine.mp.<br>8. bupivacaine hydrochloride.mp.<br>9. exparel.mp.<br>10. marcaine.mp.<br>11. marcaine hydrochloride.mp.<br>12. sensorcaine.mp.<br>13. 6 or 7 or 8 or 9 or 10 or 11 or 12<br>14. [embase clinical trials filter]<br>15. exp crossover procedure/<br>16. exp double blind procedure/<br>17. exp randomized controlled trial/<br>18. exp single blind procedure/<br>19. (random* or factorial* or crossover* or (cross adj over*) or placebo* or (double* adj blind*) or (singl* adj blind*) or assign* or allocat* or volunteer*).mp.<br>20. 15 or 16 or 17 or 18 or 19<br>21. 5 and 13 and 20<br>22. ("Levobupivacaine hydrochloride" or "L(-)-Bupivacaine" or "(S)-bupivacaine" or "L-(-)-bupivacaine" or "S(-)-bupivacaine" or "levo-(S)-bupivacaine" or "(L)-(-)-bupivacaine" or "(S)-(-)-Bupivacaine" or "l-bupivacaine").mp.<br>23. 5 or 22<br>24. 13 and 20 and 23
**Levocetirizine vs. Cetirizine**	1. ("(R)-cetirizine" or "R-levocetirizine" or "l-cetirizine").mp.<br>2. [levocetirizine vs cetirizine]<br>3. levocetirizine.mp.

© 2021 Long AS et al. *JAMA Network Open.*

	4. levocetirizine hydrochloride.mp.<br>5. levocetirizine dihydrochloride.mp.<br>6. xyzal.mp.<br>7. 3 or 4 or 5 or 6<br>8. [MeSH term deleted]<br>9. cetirizine.mp.<br>10. cetirizine hydrochloride.mp.<br>11. cetirizine hydrochloride allergy.mp.<br>12. cetirizine hydrochloride hives.mp.<br>13. zyrtec.mp.<br>14. zyrtec-d.mp.<br>15. zerivate.mp.<br>16. 8 or 9 or 10 or 11 or 12 or 13 or 14 or 15<br>17. [clinical trials]<br>18. randomized controlled trial.pt.<br>19. controlled clinical trial.pt.<br>20. randomized.ab.<br>21. placebo.ab.<br>22. drug therapy.fs.<br>23. randomly.ab.<br>24. trial.ab.<br>25. groups.ab.<br>26. 18 or 19 or 20 or 21 or 22 or 23 or 24 or 25<br>27. exp animals/ not humans.sh.<br>28. 26 not 27<br>29. [ANDing]<br>30. 7 and 16 and 28<br>31. 1 or 7<br>32. 16 and 28 and 31
**Levofloxacin vs. Ofloxacin**	1. [levofloxacin vs ofloxacin]<br>2. exp Levofloxacin/<br>3. levofloxacin.mp.<br>4. quixin.mp.<br>5. iquix.mp.<br>6. levaquin.mp.<br>7. 2 or 3 or 4 or 5 or 6<br>8. Ofloxacin/<br>9. ofloxacin.mp.<br>10. floxin.mp.<br>11. floxin otic.mp.<br>12. ocuflox.mp.<br>13. 8 or 9 or 10 or 11 or 12<br>14. [clinical trials]<br>15. randomized controlled trial.pt.<br>16. controlled clinical trial.pt.

© 2021 Long AS et al. *JAMA Network Open.*

	17. randomized.ab.<br>18. placebo.ab.<br>19. drug therapy.fs.<br>20. randomly.ab.<br>21. trial.ab.<br>22. groups.ab.<br>23. 15 or 16 or 17 or 18 or 19 or 20 or 21 or 22<br>24. exp animals/ not humans.sh.<br>25. 23 not 24<br>26. [ANDing together]<br>27. 7 and 13 and 25<br>28. ("Levofloxacin hydrate" or "Iquix hydrate" or "Levaquin hydrate" or "(S)-Ofloxacin" or "L-Ofloxacin" or "(S)-(-)-Ofloxacin" or "S-(-)-Ofloxacin" or "DR-3355" or "Levofloxacine").mp.<br>29. 7 or 28<br>30. 13 and 25 and 29
**Levoleucovorin vs. Leucovorin**	1. ("(6S)-Leucovorin" or "(6S)-Folinic acid" or "Levofolinic acid" or "L-Folinic acid" or "(S)-Leucovorin" or "l-Leucovorin" or "levo-Folinic" or "L(-)-5-Formyl-5,6,7,8-tetrahydrofolic acid" or "levofolinate" or "l-folinate l-LV" or "(6S)-5-formyltetrahydrofolate" or "levofolene" or "levo-leucovorin").mp.<br>2. ("d,l-LV" or "5-formyltetrahydrofolate" or "folinic" or "folinate").mp.<br>3. exp folinic acid/<br>4. [levoleucovorin vs leucovorin]<br>5. exp Levoleucovorin/<br>6. levoleucovorin.mp.<br>7. levoleucovorin calcium.mp.<br>8. fusilev.mp.<br>9. khapzory.mp.<br>10. 5 or 6 or 7 or 8 or 9<br>11. Leucovorin/<br>12. leucovorin.mp.<br>13. leucovorin calcium.mp.<br>14. wellcovorin.mp.<br>15. 11 or 12 or 13 or 14<br>16. [clinical trials]<br>17. randomized controlled trial.pt.<br>18. controlled clinical trial.pt.<br>19. randomized.ab.<br>20. placebo.ab.<br>21. drug therapy.fs.<br>22. randomly.ab.<br>23. trial.ab.

© 2021 Long AS et al. *JAMA Network Open.*

	24. groups.ab.<br>25. 17 or 18 or 19 or 20 or 21 or 22 or 23 or 24<br>26. exp animals/ not humans.sh.<br>27. 25 not 26<br>28. 10 and 15 and 27
**Levomilnacipran vs. Milnacipran**	1. [levomilnacipran vs milnacipran]<br>2. exp Levomilnacipran/<br>3. levomilnacipran.mp.<br>4. levomilnacipran hydrochloride.mp.<br>5. fetzima.mp.<br>6. 2 or 3 or 4 or 5<br>7. Milnacipran/<br>8. milnacipran.mp.<br>9. milnacipran hydrochloride.mp.<br>10. savella.mp.<br>11. 7 or 8 or 9 or 10<br>12. [clinical trials]<br>13. randomized controlled trial.pt.<br>14. controlled clinical trial.pt.<br>15. randomized.ab.<br>16. placebo.ab.<br>17. drug therapy.fs.<br>18. randomly.ab.<br>19. trial.ab.<br>20. groups.ab.<br>21. 13 or 14 or 15 or 16 or 17 or 18 or 19 or 20<br>22. exp animals/ not humans.sh.<br>23. 21 not 22<br>24. [anding]<br>25. 6 and 11 and 23<br>26. ("(+)-(1S,2R)-MILNACIPRAN" or "(1S,2R)-milnacipran" or "l-milnacipran").mp.<br>27. 6 or 26<br>28. 11 and 23 and 27
[a] The other database searches were adapted from these to have the appropriate subject headings, filters, etc.	

© 2021 Long AS et al. *JAMA Network Open.*

**eTable 2.** Characteristics of Included Studies												
**First Author Last Name**	**Publication Year**	**Single-enantiomer Drug**	**Journal title**	**Article type**	**Enrollment size**	**Ages included**	**Sex included**	**Allocation**	**Study Duration**	**Other drugs included in each arm**	**Additional arm(s)**	**Indication**
Ahmad	2012	Levobupivacaine	Middle east african journal of ophthalmology	Full text	150	Adult (18-65), Elderly (>65)	Both	Double-blind	Less than one day	lidocaine	none	Nerve block for peribulbar surgery
Akarsu	2011	Levobupivacaine	Turkiye klinikleri journal of medical sciences	Full text	80	Adult (18-65)	Both	Double-blind	Less than one day	fentanyl	none	Epidural nerve block for lumbar laminectomy and discectomy
Akcaboy	2011	Levobupivacaine	Journal of research in medical sciences	Full text	50	Adult (18-65), Elderly (>65)	Male	Double-blind	Less than one day	fentanyl	none	Epidural nerve block for prostatectomy
Aksu	2009	Levobupivacaine	European journal of ophthalmology	Full text	90	Adult (18-65), Elderly (>65)	Both	Single-blind	Not reported		lidocaine	Nerve block for retrobulbar surgery
Aksu	2011	Levobupivacaine	Turkiye klinikleri journal of medical sciences	Full text	120	Adult (18-65), Elderly (>65)	Both	Double-blind	Less than one day		none	Nerve block for retrobulbar surgery
Al	2012	Levobupivacaine	Middle east journal of anesthesiology	Full text	30	Adult (18-65)	Both	Single-blind	Hours		none	Nerve block for hand or forearm surgery

© 2021 Long AS et al. *JAMA Network Open.*

Anagnostopoulos	2004	Esomeprazole	Journal of clinical gastroenterology	Full text	156	Adult (18-65), Elderly (>65)	Both	Unclear	Weeks	amoxicillin; clarithromycin	none	Helicobacter pylori; Dyspepsia
Andrews	2009	Levalbuterol	The journal of pediatrics	Full text	81	Pediatric (<18)	Both	Double-blind	Hours		none	Status asthmaticus/Severe Asthma
Armstrong I	2004	Esomeprazole	Alimentary pharmacology & therapeutics	Full text	1282	Adult (18-65), Elderly (>65)	Both	Double-blind	Weeks		none	Endoscopy-Negative Reflux Disease (ENRD)
Armstrong II	2004	Esomeprazole	Alimentary pharmacology & therapeutics	Full text	693	Adult (18-65), Elderly (>65)	Both	Double-blind	Weeks		none	Endoscopy-Negative Reflux Disease (ENRD)
Armstrong III	2004	Esomeprazole	Alimentary pharmacology & therapeutics	Full text	670	Adult (18-65), Elderly (>65)	Both	Double-blind	Weeks		none	Endoscopy-Negative Reflux Disease (ENRD)
Atienzar	2008	Levobupivacaine	International journal of obstetric anesthesia	Full text	63	Adult (18-65)	Female	Double-blind	Less than one day	fentanyl	ropivacaine, fentanyl	Epidural nerve block for labor
Avci	2012	Levobupivacaine	European Urology	Abstract	80	Unclear	Male	Unclear	Less than one day		no intervention; lidocaine	Nerve block for prostate biopsy
Aydin	2009	Levobupivacaine	Anestezi dergisi	Abstract	40	Unclear	Female	Unclear	Less than one day		ropivacaine	Spinal nerve block for cesarean section
Bader	1999	Levobupivacaine	Anesthesiology	Full text	60	Adult (18-65)	Female	Double-blind	Less than one day		none	Epidural nerve block for cesarean section

© 2021 Long AS et al. *JAMA Network Open.*

Balestrieri	2003	Levobupivacaine	Anesthesiology	Abstract	19	Adult (18-65)	Female	Double-blind	Less than one day	fentanyl	none	Epidural nerve block for labor
Baliuliene	2018	Levobupivacaine	International journal of obstetric anesthesia	Full text	246	Adult (18-65)	Female	Double-blind	Less than one day	fentanyl	none	Epidural nerve block for labor
Baliuliene	2018	Levobupivacaine	International journal of obstetric anesthesia	Full text	246	Adult (18-65)	Female	Double-blind	Less than one day	fentanyl	none	Epidural nerve block for labor
Baskan	2010	Levobupivacaine	Journal of anesthesia	Full text	60	Adult (18-65), Elderly (>65)	Both	Double-blind	Less than one day		none	Nerve block for shoulder surgery
Bay-Nielsen	1999	Levobupivacaine	British journal of anaesthesia	Full text	66	Adult (18-65), Elderly (>65)	Male	Double-blind	Days		none	Nerve block for inguinal hernia repair
Beh	2011	Levocetirizine	Clinicaltrials.gov	Unpublished trial	466	Pediatric (<18), Adult (18-65)	Both	Double-blind	Days	hydrocortisone	none	Dermatitis and Eczema
Beilin	2007	Levobupivacaine	International anesthesia research society	Full text	132	Adult (18-65)	Female	Double-blind	Less than one day	fentanyl	ropivacaine, fentanyl	Epidural nerve block for labor
Bergamaschi	2005	Levobupivacaine	Brazilian journal of anesthesiology	Full text	47	Pediatric (<18), Adult (18-65)	Female	Double-blind	Less than one day	sufentanil; epinephrine	none	Epidural nerve block for cesarean section
Berger	2006	Levalbuterol	Current medical research and opinion	Full text	115	Pediatric (<18)	Both	Double-blind	Days		Placebo	Asthma

© 2021 Long AS et al. *JAMA Network Open.*

Bhatia	2014	Dexlansoprazole	Indian journal of gastroenterology	Abstract	260	Adult (18-65)	Both	Double-blind	Weeks		none	Gastroesophageal Reflux Disease
Birt	2004	Levobupivacaine	Eye	Full text	60	Adult (18-65), Elderly (>65)	Both	Double-blind	Less than one day	hyaluronidase	none	Nerve block for peribulbar surgery
Brajković	2015	Levobupivacaine	Vojnosanitetski pregled	Full text	68	Adult (18-65)	Both	Double-blind	Less than one day		lidocaine, epinephrine	Nerve block for dental surgery
Brajković	2014	Levobupivacaine	Clinical oral investigations	Full text	60	Adult (18-65)	Both	Double-blind	Days		none	Nerve block for dental surgery
Bremerich	2007	Levobupivacaine	Current medical research and opinion	Full text	60	Adult (18-65)	Female	Double-blind	Less than one day	fentanyl; sufentanil	none	Spinal block for cesarean section
Brunetti	2015	Levalbuterol	American journal of health-system pharmacy	Full text	112	Adult (18-65), Elderly (>65)	Both	Open-label	Years		none	Asthma or Chronic Obstructive Pulmonary Disease (COPD)
Burke	1999	Levobupivacaine	British journal of anaesthesia	Full text	137	Adult (18-65)	Female	Double-blind	Days		none	Epidural nerve block for labor
Burke	2002	Escitalopram	Journal of clinical psychiatry	Full text	361	Adult (18-65)	Both	Double-blind	Weeks		placebo	Major Depressive Disorder, MADRS >=22
Buyse	2006	Levobupivacaine	International journal of obstetric anesthesia	Full text	108	Adult (18-65)	Female	Double-blind	Less than one day	none; sufentanil	ropivacaine; ropivacaine, sufentanil	Epidural nerve block for labor
Cacciapuoti	2002	Levobupivacaine	Minerva anestesiologica	Full text	30	Adult (18-65), Elderly (>65)	Both	Open-label	Less than one day		ropivacaine	Nerve block for hand or wrist surgery

© 2021 Long AS et al. *JAMA Network Open.*

Can	2017	Levobupivacaine	Journal of international medical research	Full text	60	Adult (18-65), Elderly (>65)	Both	Double-blind	Less than one day		placebo	Nerve block for craniotomy
Carl	2003	Levalbuterol	The journal of pediatrics	Full text	547	Pediatric (<18)	Both	Double-blind	Not reported		none	Status asthmaticus/Severe Asthma
Casati	2002	Levobupivacaine	Journal of clinical anesthesia	Full text	30	Adult (18-65), Elderly (>65)	Both	Double-blind	Less than one day		none	Nerve block for foot surgery
Casimiro	2008	Levobupivacaine	Minerva Anesthesiology	Full text	96	Adult (18-65), Elderly (>65)	Both	Single-blind	Less than one day	fentanyl	none	Epidural nerve block for lower limb surgery
Celik	2013	Levobupivacaine	Slovenian medical journal	Full text	60	Adult (18-65)	Both	Single-blind	Less than one day	fentanyl	none	Spinal block for hip surgery
Chen	2005	Esomeprazole	World journal of gastroenterology	Full text	48	Adult (18-65), Elderly (>65)	Both	Double-blind	Weeks		none	Erosive oesophagitis
Cheng	2002	Levobupivacaine	Acta anaesthesiologica sinica	Full text	45	Adult (18-65)	Female	Double-blind	Days		none	Epidural nerve block for cesarean section
Cok	2011	Levobupivacaine	Journal of cardiothoracic and vascular anesthesia	Full text	50	Adult (18-65), Elderly (>65)	Both	Double-blind	Days		none	Epidural nerve block for thoracic surgery
Colonna	2005	Escitalopram	Current medical research and opinion	Full text	357	Adult (18-65), Elderl	Both	Double-blind	Weeks		none	Major Depressive Disorder, MADRS >=22

© 2021 Long AS et al. *JAMA Network Open.*

						y (>65)						
Compagna	2013	Levobupivacaine	BMC Surgery	Full text	80	Elderly (>65)	Both	Double-blind	Days		none	Nerve block for inguinal hernia repair
Compagna	2012	Levobupivacaine	BMC Surgery	Full text	40	Elderly (>65)	Male	Double-blind	Less than one day		none	Nerve block for inguinal hernia repair
Convery	1999	Levobupivacaine	Intensive care medicine	Abstract	112	Adult (18-65)	Female	Double-blind	Less than one day		none	Epidural nerve block for labor
Coppejans	2004	Levobupivacaine	International journal of obstetric anesthesia	Abstract	70	Adult (18-65)	Female	Double-blind	Not reported	sufentanil	none	Epidural nerve block for cesarean section
Cox	1998	Levobupivacaine	British journal of anaesthesia	Full text	75	Adult (18-65), Elderly (>65)	Both	Double-blind	Days		none	Nerve block for Hand surgery
Cox	1998	Levobupivacaine	British journal of anaesthesia	Full text	88	Adult (18-65), Elderly (>65)	Both	Double-blind	Less than one day		none	Nerve block for Lower limb surgery
Cuvas	2009	Levobupivacaine	The association of anaesthetists of great britain and ireland	Full text	50	Adult (18-65), Elderly (>65)	Male	Double-blind	Less than one day		none	Spinal nerve block for pilonidal cyst and sinus operations
Cuvas	2008	Levobupivacaine	Minerva anesthesiology	Full text	50	Adult (18-65), Elderly (>65)	Male	Double-blind	Less than one day		none	Spinal nerve block for transurethral resection
de Leeuw	2008	Levobupivacaine	Pain practice	Full text	30	Adult (18-65), Elderl	Both	Double-blind	Days	epinephrine	ropivacaine, epinephrine	Nerve block for hip surgery

© 2021 Long AS et al. *JAMA Network Open.*

						y (>65)						
De Negri	2004	Levobupivacaine	Pediatric anesthesia	Full text	55	Pediatric (<18)	Male	Single-blind	Less than one day		ropivacaine	Epidural nerve block for hypspadias repair
del-Rio-Vellosillo	2014	Levobupivacaine	Biomed research international	Full text	60	Adult (18-65)	Both	Double-blind	Weeks		none	Spinal nerve block for knee surgery
Demir	2015	Levobupivacaine	Turkish journal of anesthesia and reanimation	Full text	60	Adult (18-65)	Male	Double-blind	Less than one day		none	Epidural nerve block for inguinal hernia repair
Deniz	2013	Levobupivacaine	Revista brasileira anestesiologica	Full text	60	Pediatric (<18), Adult (18-65)	Female	Double-blind	Less than one day		none	Spinal nerve block for cesarean section
Dogan	2014	Levobupivacaine	Basic & clinical pharmacology & toxicology	Full text	60	Adult (18-65)	Female	Double-blind	Less than one day		none	Spinal nerve block for cesarean section
Donohue	2006	Levalbuterol	COPD: journal of chronic obstructive disease	Full text	154	Adult (18-65), Elderly (>65)	Both	Double-blind	Weeks		Placebo	Chronic Obstructive Pulmonary Disease (COPD)
Donohue	2008	Levalbuterol	Clinical therapeutics	Full text	479	Adult (18-65), Elderly (>65)	Both	Open-label	Days		none	Severe asthma or Chronic Obstructive Pulmonary Disease (COPD)
Duma	2005	Levobupivacaine	British journal of anaesthesia	Full text	80	Adult (18-65)	Both	Double-blind	Less than one day	clonidine; NaCl	none	Nerve block for Hand or forearm surgery
Eker	2009	Levobupivacaine	European journal of pain	Abstract	64	Unclear	Unclear	Double-blind	Days		none	Nerve block for Shoulder surgery
Erbay	2010	Levobupivacaine	Regional anesthesia	Abstract	60	Unclear	Unclear	Double-blind	Less than	fentanyl	none	Spinal nerve block for

© 2021 Long AS et al. *JAMA Network Open.*

			and pain medicine						one day			arthroscopic surgery
Erdil	2009	Levobupivacaine	The journal of the association of anaesthetists of great britain and ireland	Full text	80	Elderly (>65)	Male	Single-blind	Less than one day	fentanyl	none	Spinal nerve block for prostate surgery
Eslik	2011	Levobupivacaine	Regional anesthesia and pain medicine	Abstract	40	Adult (18-65), Elderly (>65)	Unclear	Unclear	Less than one day		none	Spinal nerve block for lower limb amputation
Faccenda	2003	Levobupivacaine	Regional anesthesia and pain medicine	Full text	62	Adult (18-65)	Female	Double-blind	Days		none	Epidural nerve block for cesarean section
Fattorini	2006	Levobupivacaine	Minerva anestesiologic	Full text	60	Adult (18-65), Elderly (>65)	Both	Double-blind	Less than one day		none	Spinal nerve block for knee or hip surgery
Frawley	2006	Levobupivacaine	Pediatric anesthesia	Full text	310	Pediatric (<18)	Both	Double-blind	Less than one day		none	Spinal nerve block for lower abdominal surgery
Garcia	2001	Levobupivacaine	Brazilian journal of anesthesiology	Full text	52	Pediatric (<18), Adult (18-65)	Female	Double-blind	Less than one day	Sufentanil	none	Epidural nerve block for cesarean section
Gautier	2003	Levobupivacaine	British journal of anaesthesia	Full text	60	Adult (18-65)	Female	Double-blind	Weeks	sufentanil	ropivacaine, sufentanil	Epidural nerve block for cesarean section
Girwalkar-Bagle	2015	Levobupivacaine	Anesthesia, pain, and intensive care	Full text	60	Pediatric (<18)	Both	Double-blind	Days	tramadol	none	Spinal nerve block for inguinal hernia repair or orchidopexy

© 2021 Long AS et al. *JAMA Network Open.*

Glaser	2002	Levobupivacaine	Anesthesia & analgesia	Full text	80	Adult (18-65), Elderly (>65)	Both	Double-blind	Less than one day		none	Spinal nerve block for hip surgery
Goldberg	1997	Levoleucovorin	Journal of clinical oncology	Full text	926	Adult (18-65), Elderly (>65)	Both	Unclear	Years	5-fluorouracil	none	Colorectal cancer
Goyal	2015	Levobupivacaine	Anesthesia essays and researches	Full text	30	Adult (18-65)	Female	Double-blind	Less than one day	fentanyl	none	Spinal nerve block for cesarean section
Gozdemir	2016	Levobupivacaine	Clinical and investigative medicine	Full text	200	Adult (18-65), Elderly (>65)	Both	Single-blind	Weeks		articaine; lidocaine	Spinal nerve block for cesarean section or minor orthopedic surgery or varicose vein or inguinal hernia repair
Gulec	2013	Levobupivacaine	Journal of international medical research	Full text	100	Elderly (>65)	Both	Unclear	Less than one day		none	Spinal nerve block for transurethral resection
Hamilos	2007	Levalbuterol	Annals of allergy, asthma & immunology	Full text	746	Pediatric (<18), Adult (18-65)	Both	Open-label	Weeks		none	Asthma
Hanania	2010	Arformoterol	Journal of chronic obstructive pulmonary disease	Full text	444	Adult (18-65), Elderly (>65)	Both	Double-blind	Weeks			Chronic Obstructive Pulmonary Disease (COPD)
Hardasmalani	2005	Levalbuterol	Pediatric emergency care	Full text	70	Pediatric (<18),	Both	Double-blind	Not reported	prednisone;	none	Acute asthma

© 2021 Long AS et al. *JAMA Network Open.*

						Adult (18-65)				ipratropium		
Hendersen	1998	Levobupivacaine	Society for obstetric anesthesia and perinatology	Abstract	137	Adult (18-65)	Female	Double-blind	Less than one day		none	Epidural nerve block for cesarean section
Honca	2014	Levobupivacaine	Acta chirurgica belgica	Full text	60	Adult (18-65)	Unclear	Double-blind	Days		placebo	Nerve block for cholecystectomy
Hong	2016	Esomeprazole	Journal of antimicrobial chemotherapy	Full text	374	Adult (18-65)	Both	Open-label	Days	Clarithromycin; Amoxicillin; Metronidazole	none	Helicobacter pylori and duodenal ulcer
Ingelmo	2007	Levobupivacaine	Pediatric anesthesia	Full text	141	Pediatric (<18)	Both	Double-blind	Less than one day		none	Epidural nerve block for abdominal and urological surgery
Ivani	2002	Levobupivacaine	Regional anesthesia and pain medicine	Full text	35	Pediatric (<18)	Both	Single-blind	Days		ropivacaine	Spinal nerve block for lower abdominal surgery
Jaiswal	2008	Levalbuterol	Annals of allergy, asthma & immunology	Abstract	97	Pediatric (<18)	Unclear	Unclear	Days		none	Asthma
Jung	2016	Levobupivacaine	American society of anesthesiologists	Abstract	60	Adult (18-65)	Female	Double-blind	Less than one day		ropivacaine	Epidural nerve block for cesarean section
Kahrilas	2000	Esomeprazole	Alimentary pharmacology & therapeutics	Full text	1960	Adult (18-65), Elderly (>65)	Both	Double-blind	Weeks		none	Reflux oesophagitis (RO)
Kalaria	2018	Levobupivacaine	Journal of clinical and diagnostic research	Full text	104	Adult (18-65)	Both	Double-blind	Less than one day		none	Epidural nerve block for inguinal hernia repair

© 2021 Long AS et al. *JAMA Network Open.*

Kalsotra	2017	Levobupivacaine	JK science	Full text	50	Adult (18-65)	Both	Double-blind	Less than one day		none	Epidural nerve block for hip and lower limb surgery
Kao	2003	Esomeprazole	Journal of the formosan medical association	Full text	100	Adult (18-65)	Both	Single-blind	Weeks		none	Reflux oesophagitis (RO), Grade A and B
Kara	2012	Levobupivacaine	Turkish journal of medical sciences	Full text	70	Adult (18-65), Elderly (>65)	Both	Double-blind	Days		none	Epidural nerve block for hip and lower limb surgery
Karaman	2009	Levobupivacaine	Saudi medical journal	Full text	40	Adult (18-65)	Both	Double-blind	Days		none	Nerve block for knee surgery
Kaya	2014	Levobupivacaine	European association of cardiothoracic anesthesiology	Abstract	40	Adult (18-65)	Unclear	Unclear	Days	fentanyl	none	Epidural nerve block post-thoracotomy
Kaya	2012	Levobupivacaine	European review for medical and pharmacological sciences	Full text	60	Pediatric (<18)	Male	Double-blind	Less than one day		none	Spinal nerve block for circumcision
Kesimci	2012	Levobupivacaine	European archives of oto-rhino-laryngology	Full text	30	Adult (18-65)	Unclear	Double-blind	Less than one day		placebo	Nerve block for sinus surgery
Kingsnorth	2002	Levobupivacaine	European journal of surgery	Full text	69	Adult (18-65), Elderly (>65)	Male	Double-blind	Days		none	Nerve block for inguinal hernia repair
Koch	2008	Levobupivacaine	Der anaesthesist	Full text	51	Adult (18-65), Elderly (>65)	Both	Single-blind	Less than one day		ropivacaine	Epidural nerve block for hip surgery
Kokulu	2014	Levobupivacaine	Anestezi dergisi	Full text	30	Adult (18-65),	Both	Unclear	Less than	fentanyl	none	Nerve block for post-thoracotomy

© 2021 Long AS et al. *JAMA Network Open.*

						Elderly (>65)			one day			
Kopacz	2000	Levobupivacaine	Regional anesthesia and pain medicine	Full text	56	Adult (18-65), Elderly (>65)	Both	Double-blind	Less than one day		none	Epidural nerve block for lower abdominal surgery
Kumar	2010	Levalbuterol	Chest	Abstract	80	Unclear	Both	Unclear	Weeks		none	Asthma
Lai	2001	Levobupivacaine	British journal of anesthesia	Full text	90	Adult (18-65), Elderly (>65)	Both	Single-blind	Less than one day	lidocaine		Nerve block for Peribulbar
Lee	2008	Levocetirizine	Pediatric allergy and immunology	Full text	50	Pediatric (<18)	Both	Double-blind	Weeks		Placebo	Perennial allergic rhinitis
Lee	2003	Levobupivacaine	Anaesthesia and intensive care	Full text	49	Adult (18-65), Elderly (>65)	Unclear	Double-blind	Less than one day		none	Spinal nerve block for urological surgery
Lepola	2003	Escitalopram	International clinical psychopharmacology	Full text	315	Adult (18-65)	Both	Double-blind	Weeks		placebo	Major Depressive Disorder, MADRS >=22
Lew	2003	Levobupivacaine	Anesthesia and intensive care	Abstract	40	Unclear	Female	Unclear	Less than one day	fentanyl	none	Spinal nerve block for cesarean section
Li	2014	Escitalopram	Annals of clinical psychiatry	Full text	577	Adult (18-65)	Both	Double-blind	Weeks		none	Major Depressive Disorder, HAM-D > 18
Lightdale	2005	Esomeprazole	Digestive diseases and sciences	Full text	1176	Adult (18-65), Elderly (>65)	Both	Double-blind	Weeks		none	Erosive oesophagitis (EO)

© 2021 Long AS et al. *JAMA Network Open.*

Liisanantti	2004	Levobupivacaine	Acta anaesthesiol scandinavica	Full text	60	Adult (18-65)	Both	Double-blind	Days		ropivacaine	Nerve block for Hand or forearm surgery
Lim	2004	Levobupivacaine	Anesthesia and analgesia	Full text	40	Adult (18-65)	Female	Double-blind	Less than one day		ropivacaine	Spinal nerve block for labor
Locatelli	2004	Levobupivacaine	British journal of anesthesia	Full text	66	Pediatric (<18)	Both	Double-blind	Less than one day		ropivacaine	Spinal nerve block for lower abdominal surgery
Luck	2008	Levobupivacaine	British journal of anesthesia	Full text	40	Adult (18-65), Elderly (>65)	Both	Double-blind	Days		ropivacaine	Spinal nerve block for lower abdominal, perineal, or lower limb surgery
Maiti	2011	Levalbuterol	Indian journal of pharmacology	Full text	60	Adult (18-65), Elderly (>65)	Both	Open-label	Weeks		none	Asthma (mild persistent)
Mantouvalou	2008	Levobupivacaine	Acta ancesthesiologica belgica	Full text	80	Adult (18-65), Elderly (>65)	Both	Unclear	Less than one day		ropivacaine	Spinal nerve block for lower abdominal surgery
McClure	1998	Levobupivacaine	Anaesthesia	Full text	50	Elderly (>65)	Both	Double-blind	Less than one day	hyaluronidase	none	Nerve block for Peribulbar
Miehlke	2003	Esomeprazole	Alimentary pharmacology & therapeutics	Full text	80	Adult (18-65), Elderly (>65)	Both	Unclear	Weeks	Clarithromycin; Metronidazole	none	Helicobacter pylori
Milgrom	2001	Levalbuterol	Journal of allergy and clinical immunology	Full text	273	Pediatric (<18)	Both	Double-blind	Days		Placebo	Asthma

© 2021 Long AS et al. *JAMA Network Open.*

Mirsadraee	2009	Levalbuterol	Tanaffos	Full text	128	Pediatric (<18), Adult (18-65), Elderly (>65)	Both	Double-blind	Hours		ipratropium	Asthma
Misirlioglu	2013	Levobupivacaine	Hippokratia	Full text	72	Adult (18-65)	Female	Double-blind	Less than one day	fentanyl	none	Spinal nerve block for cesarean section
Moore	2005	Escitalopram	International clinical psychopharmacology	Full text	294	Adult (18-65)	Both	Double-blind	Weeks		none	Severe Major Depressive Disorder, MADRS >=30
Morris	2005	Esomeprazole	Gut	Abstract	553	Unclear	Unclear	Double-blind	Weeks		none	Reflux oesophagitis (RO), Grade C and D
Mostafa	2018	Levobupivacaine	Korean journal of anesthesiology	Full text	60	Pediatric (<18)	Both	Double-blind	Days		none	Nerve block for cleft palate surgery
Nada	2012	Escitalopram	The journal of sexual medicine	Abstract	60	Unclear	Male	Double-blind	Weeks		none	Premature Ejaculation
Naithani	2017	Levobupivacaine	Clinical trials registry of India	Unpublished trial	107	Adult (18-65)	Female	Double-blind	Less than one day		none	Spinal nerve block for cesarean section
Nelson	1998	Levalbuterol	Journal of allergy and clinical immunology	Full text	287	Pediatric (<18), Adult (18-65), Elderly (>65)	Both	Double-blind	Days		placebo	Asthma
Ngamprasertwong	2005	Levobupivacaine	Journal of the medical association of thailand	Full text	61	Adult (18-65)	Female	Double-blind	Less than one day		none	Epidural nerve block for cesarean section

© 2021 Long AS et al. *JAMA Network Open.*

Novak-Jankovic	2012	Levobupivacaine	Journal of cardiothoracic and vascular anesthesia	Full text	40	Adult (18-65), Elderly (>65)	Both	Double-blind	Days	morphine	none	Nerve block for thoracic surgery
Nowak	2006	Levalbuterol	The american journal of emergency medicine	Full text	627	Adult (18-65)	Both	Double-blind	Hours	prednisone	none	Acute asthma
Ou	2011	Escitalopram	Psychopharmacology	Full text	240	Adult (18-65)	Both	Double-blind	Weeks		none	Major Depressive Disorder, HAM-D > 18
Özmen	2011	Levobupivacaine	Annals of otology, rhinology & laryngology	Full text	40	Pediatric (<18)	Both	Double-blind	Days		placebo	Nerve block for tonsillectomy
Ozturk	2015	Levobupivacaine	BMC anesthesiology	Full text	40	Adult (18-65)	Both	Double-blind	Days		placebo	Nerve block for shoulder surgery
Ozyuvaci	2012	Levobupivacaine	World institute of pain	Abstract	30	Unclear	Unclear	Double-blind	Not reported		none	Nerve block for Hand or forearm surgery
Ozyuvaci	2010	Levobupivacaine	Regional anesthesia and pain medicine	Abstract	40	Adult (18-65)	Unclear	Unclear	Not reported		none	Nerve block for Shoulder and arm surgery
Pacella	2010	Levobupivacaine	European review for medical and pharmacological sciences	Full text	120	Adult (18-65), Elderly (>65)	Both	Double-blind	Days	hyaluronidase	none	Nerve block for Peribulbar
Pacella	2013	Levobupivacaine	Clinical ophthalmology	Full text	160	Adult (18-65), Elderly (>65)	Both	Double-blind	Days	hyaluronidase	none	Nerve block for Peribulbar
Palomaki	2005	Levobupivacaine	Acta obstetricia et gynecologica scandinavica	Full text	397	Adult (18-65)	Female	Double-blind	Days		none	Nerve block for labor

© 2021 Long AS et al. *JAMA Network Open.*

Pedro	2009	Levobupivacaine	Brazilian journal of anesthesiology	Full text	50	Adult (18-65)	Both	Double-blind	Less than one day		none	Nerve block for Shoulder and arm surgery
Peng	1999	Levofloxacin	Journal of microbiology, iImmunology and infection	Full text	46	Pediatric (<18), Adult (18-65)	Both	Double-blind	Days		none	Complicated Urinary Tract Infections
Pereira	2013	Levobupivacaine	Obstetric anaesthesia	Abstract	42	Adult (18-65)	Female	Unclear	Less than one day	sufentanil	none	Epidural nerve block for cesarean section
Pinto	2016	Eszopiclone	Clinics	Full text	199	Adult (18-65)	Both	Double-blind	Weeks		none	Insomnia
Punj	2009	Levalbuterol	Indian journal of pediatrics	Full text	60	Pediatric (<18)	Both	Double-blind	Hours		none	Acute asthma
Qureshi	2005	Levalbuterol	Annals of emergency medicine	Full text	129	Pediatric (<18)	Both	Double-blind	Less than one day		none	Acute asthma
Rani	2008	Levocetirizine	International Conference of Translational Pharmacology and 41st Annual Conference of Indian Pharmacological Society	Abstract	60	Unclear	Unclear	Open-label	Weeks		none	Seasonal allergic rhinitis
Rathore	2013	Levalbuterol	International journal of pharmaceutical sciences and research	Full text	80	Adult (18-65)	Both	Double-blind	Weeks		none	Asthma
Richard	2000	Levofloxacin	Infectious diseases in clinical practice	Full text	594	Adult (18-65), Elderly (>65)	Female	Double-blind	Weeks		none	Uncomplicated Urinary Tract Infections

© 2021 Long AS et al. *JAMA Network Open.*

Richter	2001	Esomeprazole	The american journal of gastroenterology	Full text	2425	Adult (18-65), Elderly (>65)	Both	Double-blind	Weeks		none	Erosive oesophagitis (EO)
Sagir	2010	Levobupivacaine	World journal of anesthesiology	Full text	132	Adult (18-65)	Both	Double-blind	Days	fentanyl	none	Spinal nerve block for knee surgery
Sah	2005	Levobupivacaine	Journal of clinical anesthesia	Full text	67	Adult (18-65)	Female	Double-blind	Less than one day	fentanyl	none	Epidural nerve block for labor
Sahin	2011	Levobupivacaine	Current therapeutic research	Full text	53	Adult (18-65), Elderly (>65)	Male	Single-blind	Less than one day		none	Spinal nerve block for urological surgery
Sahin	2014	Levobupivacaine	European spine journal	Full text	60	Adult (18-65), Elderly (>65)	Both	Single-blind	Less than one day		none	Spinal nerve block for lumbar disc surgery
Sathitkarnmanee	2011	Levobupivacaine	Journal of the medical association of thailand	Full text	70	Adult (18-65)	Both	Double-blind	Less than one day		none	Spinal nerve block for lower abdominal and lower extremity surgery
Scheithauer	1994	Levoleucovorin	XVI International Cancer Congress	Full text	202	Adult (18-65), Elderly (>65)	Both	Unclear	Not reported	5-fluorouracil	none	Colorectal cancer
Schmitt	2005	Esomeprazole	Digestive diseases and sciences	Full text	1148	Adult (18-65), Elderly (>65)	Both	Double-blind	Weeks		none	Erosive oesophagitis (EO)
Schwab	2003	Levofloxacin	Ophthalmology	Full text	423	Pediatric (<18),	Both	Double-blind	Days		none	Bacterial Conjunctivitis

© 2021 Long AS et al. *JAMA Network Open.*

						Adult (18-65), Elderly (>65)						
Sezen	2014	Levobupivacaine	International journal of clinical and experimental medicine	Full text	68	Pediatric (<18)	Both	Double-blind	Days	tramadol	none	Spinal nerve block for inguinal hernia repair or orchidopexy
Shah	2019	Levobupivacaine	Anesthesia, pain, and intensive care	Full text	100	Adult (18-65), Elderly (>65)	Both	Double-blind	Less than one day	lignocaine, hyalurodinase	none	Nerve block for Peribulbar
Sharma I	2009	Dexlansoprazole	Alimentary pharmacology & therapeutics	Full text	2038	Adult (18-65), Elderly (>65)	Both	Double-blind	Weeks		none	Erosive Oesophagitis
Sharma II	2009	Dexlansoprazole	Alimentary pharmacology & therapeutics	Full text	2054	Adult (18-65), Elderly (>65)	Both	Double-blind	Weeks		none	Erosive Oesophagitis
Sheu	2004	Esomeprazole	Alimentary pharmacology & therapeutics	Full text	200	Adult (18-65)	Both	Single-blind	Weeks	clarithromycin; amoxicillin	none	Helicobacter pylori
Shih	2010	Levobupivacaine	World journal of surgery	Full text	106	Pediatric (<18), Adult (18-65), Elderly (>65)	Both	Single-blind	Days		placebo	Nerve block for thyroid surgery
Sierra	2005	Esomeprazole	AGA abstracts	Abstract	320	Unclear	Both	Double-blind	Weeks		none	Erosive oesophagitis (EO), Grade C and D

© 2021 Long AS et al. *JAMA Network Open.*

Singh	2018	Levobupivacaine	Korean journal of anesthesiology	Full text	100	Adult (18-65)	Unclear	Double-blind	Less than one day		none	Spinal nerve block for inguinal hernia repair
Skoner	2005	Levalbuterol	Pediatric pulmonology	Full text	161	Pediatric (<18)	Both	Double-blind	Days		Placebo	Asthma
Sundarathiti	2014	Levobupivacaine	Journal of the medical association of thailand	Full text	90	Adult (18-65)	Female	Double-blind	Days	fentanyl	none	Spinal nerve block for cesarean section
Tai	2019	Dexlansoprazole	Infection and drug resistance	Full text	202	Adult (18-65)	Both	Open-label	Weeks	Clarithromycin; amoxicillin; metronidazole	none	Helicobacter pylori
Tan	2008	Levobupivacaine	Annual Scientific Meeting of the Australian and New Zealand College of Anaesthetists	Abstract	41	Adult (18-65)	Female	Double-blind	Less than one day	fentanyl	none	Spinal nerve block for cesarean section
Tanaka	2005	Levobupivacaine	Revista brasileira de anestesiologia	Full text	54	Adult (18-65)	Unclear	Double-blind	Less than one day	fentanyl	enantiomeric excess bupivacaine	Epidural nerve block for lower abdominal surgery
Tembe	2011	Armodafinil	Neurology research international	Full text	211	Adult (18-65)	Both	Double-blind	Weeks			Excessive sleepiness associated with shift work sleep disorder (SWSD)
Thakore	2018	Levobupivacaine	Journal of obstetric anaesthesia and critical care	Full text	90	Adult (18-65)	Female	Double-blind	Less than one day	fentanyl	none	Spinal nerve block for pregnancy termination and sterilization
Ture	2019	Levobupivacaine	Indian journal of anaesthesia	Full text	60	Adult (18-65)	Both	Double-blind	Days		none	Spinal nerve block for lower abdominal or

© 2021 Long AS et al. *JAMA Network Open.*

												lower limb surgery
Turkmen	2012	Levobupivacaine	Middle east journal of anesthesia	Full text	50	Adult (18-65)	Female	Single-blind	Less than one day	fentanyl	none	Spinal nerve block for cesarean section
Ulger	2015	Levobupivacaine	Regional anesthesia and pain medicine	Abstract	60	Unclear	Unclear	Double-blind	Less than one day		none	Nerve block for thoracic surgery
Urbanek	2003	Levobupivacaine	Regional anesthesia	Full text	60	Adult (18-65), Elderly (>65)	Both	Double-blind	Days		none	Nerve block for Lower limb surgery
Uzuner	2011	Levobupivacaine	Journal of medicine and scientific research	Full text	50	Adult (18-65), Elderly (>65)	Both	Double-blind	Less than one day	fentanyl	none	Epidural nerve block for abdominal surgery
Van Buskirk	1999	Levofloxacin	Investigative ophthalmology & visual science	Abstract	46	Pediatric (<18)	Unclear	Unclear	Weeks		none	Bacterial Conjunctivitis; Blepharoconjunctivitis
Vanna	2006	Levobupivacaine	Journal of the medical association of thailand	Full text	70	Adult (18-65), Elderly (>65)	Both	Double-blind	Days		none	Spinal nerve block for transurethral endoscopy
Varshney	2016	Levobupivacaine	Anesthesia, pain, and intensive care	Full text	60	Adult (18-65), Elderly (>65)	Both	Double-blind	Days	fentanyl	none	Nerve block for knee surgery
Veldhuyzen van Zanten	2000	Esomeprazole	Alimentary pharmacology & therapeutics	Full text	448	Adult (18-65), Elderly (>65)	Both	Double-blind	Weeks	clarithromycin; amoxicillin	none	Helicobacter pylori and duodenal ulcer

© 2021 Long AS et al. *JAMA Network Open.*

Vercaiterem	2001	Levobupivacaine	Anesthesia and analgesia	Full text	80	Adult (18-65)	Female	Double-blind	Less than one day	sufentanil; epinephrine	none	Spinal nerve block for labor
Vercauteren	2009	Levobupivacaine	International journal of obstetric anesthesia	Abstract	100	Adult (18-65)	Female	Double-blind	Less than one day	sufentanil	none	Spinal nerve block for cesarean section
Vives	2019	Levobupivacaine	BMC anesthesiology	Full text	58	Elderly (>65)	Both	Double-blind	Days	fentanyl	none	Spinal nerve block for hip surgery
Wigal	2004	Dexmethylphenidate	The journal of the american academy of child & adolescent psychiatry	Full text	90	Pediatric (<18)	Both	Double-blind	Weeks		placebo	Attention Deficit Hyperactivity Disorder (ADHD)
Yadev	2016	Levobupivacaine	Anesthesia, pain, and intensive care	Full text	100	Adult (18-65), Elderly (>65)	Both	Double-blind	Less than one day		none	Spinal nerve block for lower abdominal surgery
Yavuz	2014	Levobupivacaine	Pakistan journal of medical science	Full text	60	Adult (18-65), Elderly (>65)	Both	Unclear	Days		none	Nerve block for knee surgery
Yevtushenk	2007	Escitalopram	Clinical therapeutics	Full text	330	Adult (18-65)	Both	Double-blind	Weeks		none	Major Depressive Disorder, MADRS >=25
Yildirim Ar	2018	Levobupivacaine	Revista brasileira de anesthesiologia	Full text	50	Adult (18-65), Elderly (>65)	Both	Double-blind	Days		none	Nerve block for cholecystectomy
Zheng	2009	Esomeprazole	World journal of gastroenterology	Full text	136	Adult (18-65), Elderly (>65)	Both	Unclear	Weeks		Lansoprazole; Pantoprazole	Reflux oesophagitis (RO)

© 2021 Long AS et al. *JAMA Network Open.*

eTable 3. Results of Efficacy and Safety Comparisons

	RCTs directly comparing safety of single-enantiomer and racemic drug, No. (%)												
**Results of Comparisons**	**Arformoterol vs. Formoterol**	**Armodafinil vs. Modafinil**	**Dexlansoprazole vs. Lansoprazole**	**Dexmethylphenidate vs. Methylphenidate**	**Escitalopram vs. Citalopram**	**Esomeprazole vs. Omeprazole**	**Eszopiclone vs. Zopiclone**	**Levalbuterol vs. Albuterol**	**Levobupivacaine vs. Bupivacaine**	**Levocetirizine vs. Cetirizine**	**Levofloxacin vs. Ofloxacin**	**Levoleucovorin vs. Leucovorin**	**Total**
**Analyses of efficacy endpoints**	**1**	**1**	**4**	**1**	**8**	**17**	**1**	**16**	**121**	**3**	**4**	**2**	**179**
Single-enantiomer favored based on primary endpoint(s)	0 (0)	0 (0)	2 (50)	0 (0)	2 (25)	6 (35)	0 (0)	4 (25)	8 (6.6)	0 (0)	1 (25)	0 (0)	23 (12.8)
Single-enantiomer favored based on secondary endpoint(s)	1 (100)	0 (0)	0 (0)	0 (0)	2 (25)	2 (12)	1 (100)	8 (50)	23 (19.0)	1 (33)	0 (0)	0 (0)	38 (21.2)
Neither favored	0 (0)	1 (100)	2 (50)	1 (100)	4 (50)	8 (47)	0 (0)	3 (19)	76 (62.8)	1 (33)	3 (75)	2 (100)	101 (56.4)
Racemic favored based on secondary endpoint(s)	0 (0)	0 (0)	0 (0)	0 (0)	0 (0)	0 (0)	0 (0)	1 (6)	9 (7.4)	1 (33)	0 (0)	0 (0)	11 (6.1)
Racemic favored based on primary endpoint(s)	0 (0)	0 (0)	0 (0)	0 (0)	0 (0)	1 (6)	0 (0)	0 (0)	5 (4.1)	0 (0)	0 (0)	0 (0)	6 (3.4)
**Analyses of safety end points**	**1**	**1**	**2**	**1**	**5**	**10**	**1**	**14**	**82**	**2**	**3**	**2**	**124**
Single-enantiomer favored	0 (0)	0 (0)	0 (0)	0 (0)	1 (20)	0 (0)	0 (0)	4 (29)	12 (14.6)	0 (0)	0 (0)	0 (0)	17 (13.7)
Neither favored	0 (0)	1 (100)	2 (100)	1 (100)	4 (80)	10 (100)	1 (100)	10 (71)	67 (81.7)	2 (67)	3 (100)	2 (100)	103 (83.1)
Racemic favored	1 (100)	0 (0)	0 (0)	0 (0)	0 (0)	0 (0)	0 (0)	0 (0)	3 (3.7)	0 (0)	0 (0)	0 (0)	4 (3.2)

© 2021 Long AS et al. *JAMA Network Open.*

eTable 4. Efficacy End Point Classification for Randomized Clinical Trials Favoring Single-Enantiomer or Racemic Drugs

	RCTs directly comparing efficacy of single-enantiomer and racemic drug, No. (%)													
**Classification of results**	**Classification of end points[a]**	**Arformoterol vs. Formoterol**	**Armodafinil vs. Modafinil**	**Dexlansoprazole vs. Lansoprazole**	**Dexmethylphenidate vs. Methylphenidate**	**Escitalopram vs. Citalopram**	**Esomeprazole vs. Omeprazole**	**Eszopiclone vs. Zopiclone**	**Levalbuterol vs. Albuterol**	**Levobupivacaine vs. Bupivacaine**	**Levocetirizine vs. Cetirizine**	**Levofloxacin vs. Ofloxacin**	**Levoleucovorin vs. Leucovorin**	**All**
**Single-enantiomer favored based on primary endpoint(s)**	Clinical outcome	0 (0)	0 (0)	0 (0)	0 (0)	0 (0)	0 (0)	0 (0)	2 (50)	3 (38)	0 (0)	0 (0)	0 (0)	5 (22)
	Clinical scale	0 (0)	0 (0)	0 (0)	0 (0)	2 (100)	1 (17)	0 (0)	1 (25)	3 (38)	0 (0)	0 (0)	0 (0)	7 (30)
	Surrogate	0 (0)	0 (0)	2 (100)	0 (0)	0 (0)	5 (83)	0 (0)	1 (25)	2 (25)	0 (0)	1 (100)	0 (0)	11 (48)
	**Total**	**0**	**0**	**2**	**0**	**2**	**6**	**0**	**4**	**8**	**0**	**1**	**0**	**23**
**Single-enantiomer favored based on secondary endpoint(s)**	Clinical outcome	0 (0)	0 (0)	0 (0)	0 (0)	0 (0)	1 (50)	1 (100)	3 (38)	10 (43)	0 (0)	0 (0)	0 (0)	15 (39)
	Clinical scale	0 (0)	0 (0)	0 (0)	0 (0)	2 (100)	0 (0)	0 (0)	1 (12)	12 (52)	1 (100)	0 (0)	0 (0)	16 (42)
	Surrogate	1 (100)	0 (0)	0 (0)	0 (0)	0 (0)	1 (50)	0 (0)	4 (50)	1 (4)	0 (0)	0 (0)	0 (0)	7 (18)
	**Total**	**1**	**0**	**0**	**0**	**2**	**2**	**1**	**8**	**23**	**1**	**0**	**0**	**38**
**Racemic favored based on secondary endpoint(s)**	Clinical outcome	0 (0)	0 (0)	0 (0)	0 (0)	0 (0)	0 (0)	0 (0)	1 (100)	2 (22)	0 (0)	0 (0)	0 (0)	3 (27)
	Clinical scale	0 (0)	0 (0)	0 (0)	0 (0)	0 (0)	0 (0)	0 (0)	0 (0)	6 (67)	1 (100)	0 (0)	0 (0)	6 (55)
	Surrogate	0 (0)	0 (0)	0 (0)	0 (0)	0 (0)	0 (0)	0 (0)	0 (0)	1 (11)	0 (0)	0 (0)	0 (0)	2 (18)
	**Total**	**0**	**0**	**0**	**0**	**0**	**0**	**0**	**1**	**9**	**1**	**0**	**0**	**11**
**Racemic favored based on primary endpoint(s)**	Clinical outcome	0 (0)	0 (0)	0 (0)	0 (0)	0 (0)	0 (0)	0 (0)	0 (0)	3 (60)	0 (0)	0 (0)	0 (0)	3 (50)
	Clinical scale	0 (0)	0 (0)	0 (0)	0 (0)	0 (0)	0 (0)	0 (0)	0 (0)	2 (40)	0 (0)	0 (0)	0 (0)	2 (33)
	Surrogate	0 (0)	0 (0)	0 (0)	0 (0)	0 (0)	1 (100)	0 (0)	0 (0)	0 (0)	0 (0)	0 (0)	0 (0)	1 (17)
	**Total**	**0**	**0**	**0**	**0**	**0**	**1**	**0**	**0**	**5**	**0**	**0**	**0**	**6**

[a] If multiple endpoints favored single-enantiomer or racemic with different classifications, the classification was made based on the order clinical outcome > clinical scale > surrogate (eg. clinical scale and surrogate would be classified as clinical scale).

© 2021 Long AS et al. *JAMA Network Open.*

eTable 5. Descriptions of Safety End Points for Randomized Clinical Trials Favoring Single-Enantiomer or Racemic Drugs

	Endpoint description (No. of RCTs in which single-enantiomer or racemic drug was superior for that endpoint)				
	**Drug comparison**	**Arformoterol vs Formoterol**	**Escitalopram vs Citalopram**	**Levalbuterol vs Albuterol**	**Levobupivacaine vs Bupivacaine**
**Favors single-enantiomer drug**	**Total number of RCTs**	0	1	4	12
	**Descriptions**		Frequency of any adverse event (n=1)	Heart rate (n=4)<br>Serum potassium (n=2)<br>β-mediated adverse effects (n=1)<br>Shaky hands (n=1)<br>Nervousness (n=1)<br>Trouble concentrating (1)	Blood pressure or hypotension (n=6)<br>Heart rate or bradycardia (n=3)<br>Frequency of any adverse event (n=1)<br>Nausea (n=1)<br>Neurological complications (n=1)<br>Perineal squeezing (n=1)<br>PR interval prolongation (n=1)<br>Supraventricular arrythmia (n=1)<br>Urinary retention (n=1)
**Favors racemic drug**	**Total number of RCTs**	1	0	0	3
	**Descriptions**	Frequency of event-based COPD exacerbations (n=1)			QT_c interval prolongation (n=1)<br>Intraoperative complications (n=1)<br>Mean arterial pressure (n=1)<br>Shivering (n=1)<br>Complications in PACU (n=1)<br>Bradycardia (n=1)

© 2021 Long AS et al. *JAMA Network Open.*
